# Per- and Polyfluoroalkyl Substances in Urine Samples from Eight-Year-Old Children Living in Northwest Spain

**DOI:** 10.3390/molecules31050900

**Published:** 2026-03-09

**Authors:** Arianna Bautista, Guillermo Fernandez-Tardon, Marta M. Rodríguez-Suárez, Adonina Tardon, Natalia Bravo, Mercè Garí, Joan O. Grimalt, Marta Llorca, Marinella Farré

**Affiliations:** 1 ON-HEALTH Group, Institute of Environmental Assessment and Water Research (IDAEA-CSIC), 08034 Barcelona, Spain; arianna.bautista@idaea.csic.es; 2 Department of Chemical Engineering and Analytical Chemistry, University of Barcelona (UB), 08028 Barcelona, Spain; 3 Health Research Institute of Asturias (ISPA), Camino del Hospital s/n, 33011 Oviedo, Spain; gfernanta@gmail.com (G.F.-T.); rodriguezsmarta@uniovi.es (M.M.R.-S.); 4 Faculty of Medicine, University Nebrija, 28040 Madrid, Spain; atardon@nebrija.es; 5 Geochemistry and Pollution Group, Institute of Environmental Assessment and Water Research (IDAEA-CSIC), 08034 Barcelona, Spain; natalia.bravo@idaea.csic.es (N.B.); merce.gari@idaea.csic.es (M.G.); joan.grimalt@idaea.csic.es (J.O.G.)

**Keywords:** per- and polyfluoroalkyl substances, mass spectrometry, human matrices, urine, human biomonitoring, children cohort

## Abstract

Per- and polyfluoroalkyl substances (PFAS) are synthetics prized for their chemical stability and functionality. Legacy PFAS such as perfluorooctanesulfonic acid (PFOS) and perfluorooctanoic acid (PFOA) have been phased out due to their persistence and toxicity. This study assessed exposure to both legacy and emerging PFAS in 281 urine samples from 8-year-old children participating in the (*Infancia y Medio Ambiente*) INMA Asturias birth cohort (northwest Spain), a region with a strong industrial background. Dietary and household information was collected via questionnaires, and urine samples were analysed using ultra-high-performance liquid chromatography (UHPLC) coupled with high-resolution mass spectrometry (HRMS) with full-scan acquisition in independent all-ion fragmentation mode. A suspected screening approach was applied to discover previously unreported PFAS and expand the detectable chemical profile, complemented by targeted analysis of 29 compounds selected for their persistence and regulatory relevance. Among them, 17 compounds were confirmed and quantified. The combined targeted and suspect-screening approach also identified novel PFAS, including fluorotelomer carboxylic acids, demonstrating the value of LC-HRMS for detecting unregulated compounds. Emerging PFAS showed the highest detection frequencies and concentrations: trifluoroacetic acid (TFA) and hexafluoropropylene oxide dimer acid (HFPO-DA, GenX) were detected in 63% and 27% of samples, respectively, with GenX reaching 10.1 ng/mL, whereas PFOA and PFOS were detected less frequently (8.5% and 3.2%) and at concentrations below 1 ng/mL, highlighting the need for epidemiological studies to achieve comprehensive PFAS exposure assessments. Associations with dietary habit exposure estimates point to dairy, protein-rich foods, vegetables, and drinking water as the main contributors.

## 1. Introduction

Per- and polyfluoroalkyl substances (PFAS) are synthetic compounds in which fluorine atoms entirely or partly replace hydrogen along the carbon chain. The carbon–fluorine bond confers high stability. Their unique physicochemical properties, including polymer-forming capacity, water and grease repellence, and high resistance to degradation, have led to its wide use in industrial and consumer products, such as coatings for textiles, leather, and paper, fire-fighting foams, paints, and pesticide formulations [1]. Due to their persistency, ubiquitous occurrence, bioaccumulation [2] in organisms and humans [3,4], biomagnification in food webs [5], and adverse effects on the environment and humans [6], the production of legacy compounds such as perfluorooctane sulfonic acid (PFOS) and perfluorooctanoic acid (PFOA) was discontinued [7].

PFAS exposure has been linked to multiple adverse health effects, including immune dysfunction [8], thyroid and sex hormone disruption [9,10,11], liver toxicity [12,13], renal effects [14], endocrine, neurotoxic, and developmental outcomes [15,16,17], and childhood neurodevelopmental disorder [18]. Legacy PFAS remain detectable in humans and their body burdens are beginning to decline [19], but much less is known about exposure to replacement PFAS, despite early evidence of human accumulation and the toxicity of emerging compounds [20]. Nevertheless, early evidence has reported human exposure, accumulation, and potential risks associated with emerging PFAS. One study investigating PFAS in household environments and human samples found that trifluoroacetic acid (TFA) was the predominant compound detected in most of the samples [20]. In another pilot study, 23 plasma samples from glioma patients were analysed for 17 PFAS, including emerging alternatives, revealing that some newer congeners may exert effects similar to legacy congeners. For example, 6:2 chlorinated polyfluorinated ether sulfonate (6:2 Cl-PFESA) exhibited brain accumulation potential like PFOS [21]. This knowledge gap is compounded by the diversity of PFAS structures [22], their varying toxicokinetics [11], the analytical challenges of highly polar PFAS [23], and the presence of precursor compounds that can transform into PFAS in vivo and in the environment [24,25]. Consequently, human exposure to many emerging PFAS remains poorly characterised, particularly in vulnerable populations such as children.

Asturias (NW Spain) has historical PFAS-related industrial activity, but very limited biomonitoring data compared with other Spanish regions. Although systematic environmental and human biomonitoring remains incomplete across Spain, PFAS have been detected throughout European environments and drinking water systems, and regional data gaps are widely recognised as a barrier to understanding local exposure levels. More than 23,000 sites across Europe have been identified as contaminated by PFAS or likely to be contaminated due to past or present industrial activity, including facilities and presumptive sites related to manufacturing and aqueous film-forming foam (AFFF) use, with a number of these located in northern Spain (the “Map of Forever Pollution” showing suspected contamination clusters across EU member states including Spain). Asturias has historically hosted metallurgical, chemical, energy, and manufacturing industries, particularly concentrated along coastal and riverine corridors, where PFAS have been widely used for surface treatments, corrosion protection, lubricants, and industrial coatings, types of industrial applications widely recognised as potential PFAS sources in contaminated soils and waters. In addition, the historical and ongoing use of AFFF at industrial facilities, ports, and emergency response sites represents a well-documented source of PFAS release to soils and surface waters, with subsequent infiltration into groundwater used for drinking water supply, consistent with broader European hotspot observations linked to firefighting foams and industrial wastes [26]. Industrial wastewater discharges and the insufficient removal of PFAS in wastewater treatment plants may further contribute to environmental loading, as PFAS are poorly retained by conventional treatment processes and can be redistributed via effluents and sludge, a mechanism highlighted in the EU assessments of PFAS persistence in water systems. The combination of industrial point sources, legacy contamination in soils, and hydrological connectivity between industrial areas, river basins, and aquifers increases the likelihood of PFAS transport into drinking water sources in Asturias, even in areas distant from active industrial operations, underscoring the importance of considering historical industrial land use and the need for enhanced regional monitoring and risk assessment. Moreover, although urine is traditionally considered less suitable for monitoring long-chain PFAS, it is increasingly recognised as a valuable matrix for detecting short-chain and highly polar PFAS. Its non-invasive collection, ability to provide larger sample volumes, and compatibility with ultra-trace quantification make it particularly well-suited for tracking more polar and mobile compounds [27,28,29].

This study aimed to (i) quantify and characterise exposure to both legacy and emerging PFAS (ultrashort-, short-chain, and replacement PFAS) in 8-year-old children living in an understudied, industrially influenced region of Spain by UHPLC-HRMS in full-scan data-independent acquisition, using all ion fragmentation (DIA/AIF) mode; (ii) perform a statistical analysis for the discrimination of the PFAS exposure based on sex and diet, and (iii) evaluate urine as a biomatrix for detecting emerging PFAS and assessing children’s exposure profiles during a critical neurodevelopmental period [30,31].

## 2. Results and Discussion

### 2.1. PFAS in Urine

Seventeen of the twenty-nine compounds that were selected for target analysis were confirmed and quantified in the urine samples of children aged 8 years. The PFAS concentrations are reported in Table S1 of the Supplementary Materials (SM), and an example of an extracted ion chromatogram (XIC) of the spiked non-CRM is shown in Figure S1 in the SM. From the total, 97% of the samples (272) presented PFAS contamination, with at least one compound being detected in each sample. Creatinine normalisation was applied to urinary concentrations to correct for differences in urine dilution due to hydration status, thereby improving comparability between samples. Table 1 summarises the detection frequencies, and the maximum, minimum, median, and mean concentrations with and without creatinine normalisation.

As listed in Table 1, the more frequently detected compound was TFA (63%) in the samples, followed by hexafluoropropylene oxide dimer acid (HFPO-DA), also known as GenX (27%). The highest concentrations corresponded to Gen X with a maximum concentration of 8.572 ng/mL (10.132 ng/mL, normalised concentration by creatinine) while TFA was found in a maximum concentration of 6.397 ng/mL (7.561 ng/mL, normalised value by creatinine). It is noteworthy that trifluoro methane sulfonic acid (TFMS) was only quantified in 3.2% of the samples. TFMS was reported in the environment for the first time in 2016 [32], and it is classified as a highly persistent and highly mobile compound that, due to its high polarity, can affect aquatic reservoirs [33,34,35]. Notwithstanding, there is a low amount of data reporting this compound due to the difficulties in its analysis [36].

The present findings are consistent with those of a recent study on PFAS levels in urine from the general adult population in the United States, which reported elevated levels of ultrashort and short-chain perfluoroalkyl acids, with a maximum concentration of 290 ng/mL for TFA and a detection frequency of 31% [20]. In the present study, the frequency of detection of TFA was higher; however, the maximum concentrations were much lower, as expected, given that the participants were 8-year-old children instead of adults.

Similarly, a previous study [37] analysing 2682 spot urine samples collected from 2013 to 2014 by the National Health and Nutrition Examination Survey (NHANES) subsample of six-year-old participants in the U.S. investigated PFAS from C4 to longer-chain compounds, including some considered substitute PFAS, such as GenX. That study found PFBA in 13% of the population, with detected concentrations ranging from 0.7 ng/mL to 3.4 ng/mL, comparable to the present study, in which PFBA was detected in 15.6% of samples, with detected levels ranging from 0.033 ng/mL to 3.236 ng/mL (0.039–3.825 ng/mL, creatinine normalised). However, the frequency of GenX detection differed notably. While it was found in only 1.2% of the samples in the NHANES study (0.070–3.2 ng/mL), it was above the method’s limit of detection in 27% of the samples in the current one, with detected concentrations ranging from 0.244 to 8.572 ng/mL (0.288–10.132 ng/mL, creatine normalised). At the same time, the concentrations reported in the present study are of a similar order to those previously reported in NHANES. Still, the frequency of detection was much higher, but the method limit of detection (MLOD) here was much lower, which can significantly influence the detection rate. The sources of these shorter chain compounds and replacement compounds perhaps reflected the influence of nearby industrial activity, not just producing but also using materials containing PFAS. The high mobility and polarity of these compounds can influence the contamination of tap water sources. However, since no analysis of tap water was included in this study, this information should be considered cautiously.

In the present study, PFHxA was also among the most frequently detected compounds (26% of the samples), with concentrations ranging from the method’s limit of quantification (MLOQ) to 2.893 ng/mL (3.420 ng/mL, creatinine normalised). Regarding C8 congeners, despite the discontinuation of PFOS and PFOA productions, these compounds remained in some samples due to their high persistence. As a result, they were still detected, albeit infrequently (3.2% for PFOS and 8.5% for PFOA) at low concentrations, ranging from the MLOQ to 0.680 (0.804 ng/mL creatinine normalised) for PFOS and from the MLOQ to 0.562 ng/mL (0.664 ng/mL creatinine normalised) for PFOA. In general, our results agree with previous studies reporting the stronger binding to serum proteins (e.g., albumin) [38] of sulfonic compounds compared to their carboxylic counterparts, leading to slower renal clearance and longer biological half-lives of sulfonic compounds [39]. Moreover, the average renal clearance rates for ultrashort and shorter chain compounds such as TFA, PFPrA, and PFBA have been estimated to be 7.3 mL/kg/day, 1.02 mL/kg/day, and 21 mL/kg/day, respectively. These rates were 10 and 1000 times higher than those reported for C8 compound PFOA (0.29 mL/kg/day) and PFOS (0.045 mL/kg/day) [40]. As a result, the corresponding average half-lives of the ultrashort and short-chain congeners ranged from 4 to 62 days, which was substantially shorter than those for long-chain compounds, ranging from 646 to 1533 days. The higher water solubility of shorter and ultrashort-chain congeners and their lower biological persistence, combined with the notably higher water solubility [41] of the short-chain compounds (0.35–9.7 × 10^5^ mg/L) compared to their long-chain analogues (ranging from 4.7 × 10^−8^ to 0.21 mg/L), likely accounts for the more frequent detection of ultrashort- and short-chain PFAS in urine samples, as was also shown in a previous study [40].

### 2.2. Differences Among Sex

**Figure 1:**
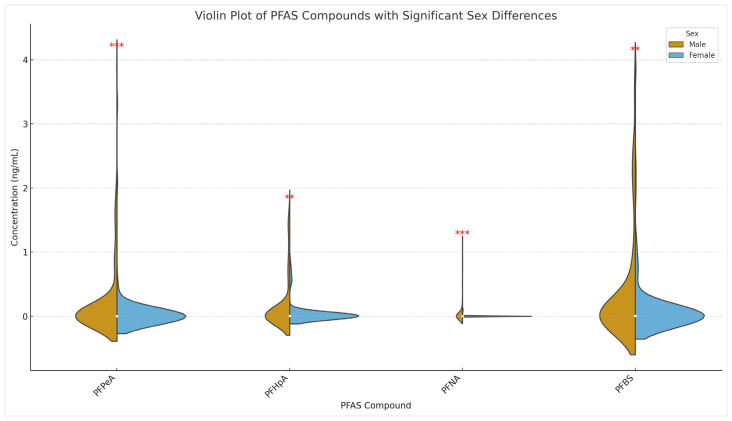
Violin plot combining the boxplot and the kernel density plot, for a comprehensive view of the data distribution of PFAS concentrations by sex. Only compounds with significant differences are presented. ** *p* < 0.01 (highly significant); *** *p* < 0.001 (very highly significant).

Regarding the potential differences of PFAS concentrations between boys and girls, the Shapiro–Wilk test for normality was first used to determine whether the data followed normal distributions. Since non-normal distributions were found, a non-parametric statistical test, the Mann–Whitney U test, also known as the Wilcoxon rank sum test, was used to assess whether there were significant differences between boys and girls. As was expected, no significant differences were obtained, in general, because the participants were children, except for PFBS (*p*-value 0.000937), PFPeA (*p*-value 0.000956), PFNA (*p*-value 0.001338), and PFHpA (*p*-value 0.002269), with *p*-values inferior to 0.05, indicating that the differences can be considered significant. Figure 1 presents the violin plot combining the boxplot with the kernel density plot, thus offering a comprehensive view of the data distribution. Although median concentrations appeared similar, the distribution patterns between sexes differed markedly. One group exhibited a sharper density peak around central values, while the other displayed a broader spread. Based on existing PFAS exposure studies, the wider dispersion along the x-axis, particularly evident in female concentration profiles, is attributed primarily to physiological differences rather than the presence of multiple metabolites within the same PFAS class. It is important to note that perfluorinated compounds such as PFOA, PFOS, and PFHxS are not chemically metabolised in the human body due to the strong carbon–fluorine (C–F) bond. As a result, these compounds tend to bioaccumulate in tissues such as blood, liver, and kidneys.

In previous studies, in general, higher differences in the accumulation were shown in post-puberty ages, where females have an extra excretion route through menstruation. However, it is challenging to compare with other studies because, in general, accumulation in serum is performed, and here, clearance rates through urine are presented. On the other hand, compounds showing significant differences could be due to different ingestion rates between boys and girls, or sex-dependent differences in the expression of metabolising enzymes and transcription factors, such as HNF4α, which regulate these enzymes, contributing to differences in how the body processes and eliminates chemicals like PFAS [42]. This also highlights the need to investigate the exposure routes of new replacement compounds and shorter-chain PFAS in depth, in combination with their clearance rates.

### 2.3. Relationships Among PFAS Exposures Through Food and Drinks

As can be seen in Table S1 of the Supplementary Materials, the complete diet was characterised for each participant. The different foods were grouped, and relationships among groups of foods that were previously characterised and identified as potential sources of PFAS through diet in other studies were analysed. The food groups established were dairy products, egg ingesta, protein foods (including fish, meat, and processed meat foods), vegetables and fruits, soft drinks, tap water, and bottled water.

**Figure 2:**
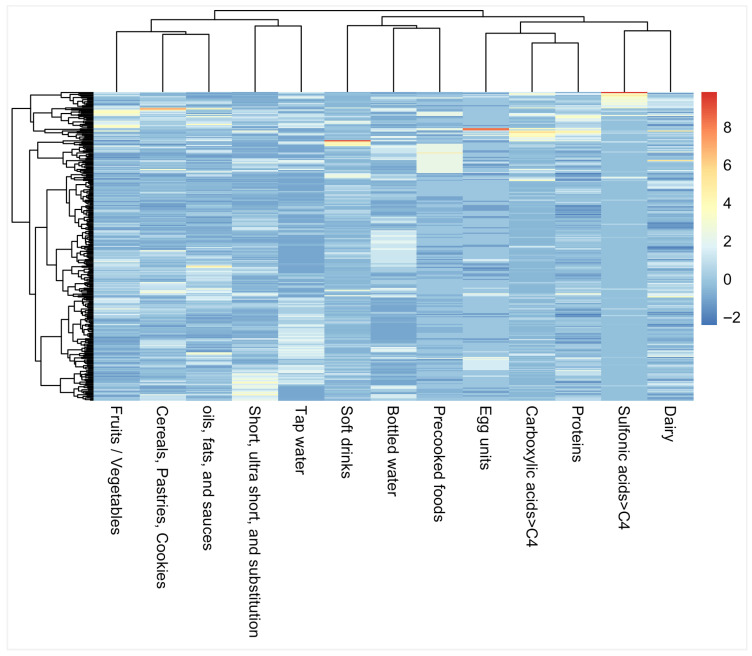
Hierarchical cluster analysis for the normalised values by the Ward clustering method and the Euclidean distance to show grouping associations between the amounts and type of foodstuff and PFAS groups in urine.

Figure 2 shows the hierarchical cluster analysis for the normalised values (Z-score) using the Ward clustering method and the Euclidean distance to obtain compact and homogeneous clusters.

As can be seen, the first group was composed of fruits and vegetables, cereals, pastries, and cookies, and oils, fats and sauces, which are variables that covariate together, meaning that individuals who consume more of one tend to consume more of the others. This represents a general dietary pattern, but these consumptions are not related to PFAS in urine. The second group was composed of short, ultrashort chain and substitution compounds that correlate with tap water. The third group was composed of soft drinks, bottled water, and precooked foods. Another group was composed of high-protein foods, eggs, and long-chain perfluoro carboxylic acids, and the last group of dairy products and long-chain perfluoro sulfonic acids. Considering the groups of individuals (left), the first cluster was composed of individuals mostly showing blue values (negative Z-scores), which means a low influence of PFAS exposure through food intake and low exposure in general. The second groups corresponded to a higher dietary Z-score, but this did not drive a higher level in urine. The third group was composed of individuals that appeared to have environmental PFAS exposure, not necessarily only linked to diet, probably due to diffuse sources such as dust ingestion, which has been shown to be another potential source of human exposure [43].

**Figure 3:**
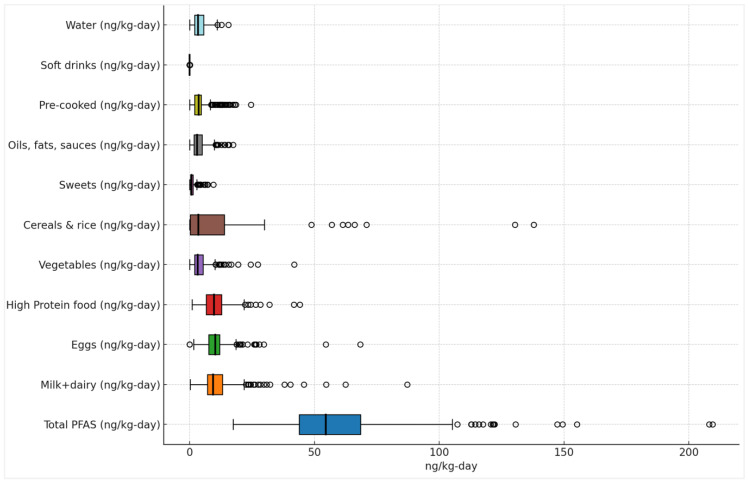
Boxplot of the influence of the different food groups according to the reported diet.

In contrast, the daily exposure estimated from the dietary composition reported by participants, combined with previously published data on long-chain PFAS concentrations in various food groups [44,45,46], resulted in a median intake of 55 ng/kg-day, with values of 18 ng/kg-day at the 5th percentile (P5), and 113 ng/kg-day at the 95th percentile (P95). As shown in Figure 3, milk and dairy products, high-protein foods such as fish and meat, eggs, and the combined group of cereals, bread, vegetables, and fruit were the primary contributors to overall exposure.

These findings align with findings reported by the European Food Safety Authority (EFSA) [47], which analysed data from 19 dietary surveys that were conducted across Europe, in order to assess exposure to 17 PFAS compounds. These included PFBA, PFPeA, PFHxA, PFHpA, PFOA, PFNA, PFDA, PFUnDA, PFDoDA, PFTrDA, PFTeDA, PFBS, PFHxS, PFHpS, PFOS, PFDS, and FOSA in children aged between 4 and 10 years. EFSA reported P95 exposure levels of 183 ng/kg body weight/day and P5 values of 4.0 ng/kg-day.

The current limitation of these studies is the lack of data regarding short and ultrashort chain compounds, substitute compounds, and fluorotelomers. Conversely, urinary concentrations of long-chain PFAS do not seem to be reliable indicators of exposure, which is likely due to the limited renal clearance. Alternative matrix, such as serum, or other excretion routes such as faeces, may offer a more accurate assessment of human exposure to long-chain PFAS. However, for short-chain compounds, urinary measurements can be considered as valid indicators of exposure, which is supported by recent findings [20].

### 2.4. PFAS Suspect Screening in Urine

A total of 281 urine samples from 8-year-old children were analysed. The identification approach was based on the workflow from Schymanski et al. [48], with some modifications. Level 1 corresponds to the confirmed structure by comparison with a reference standard. Tentative level 2 corresponds to tentative identification of a compound The structure is highly likely based on spectral library match or diagnostic evidence, but no reference standard was used and instead achieved through MS^2^ spectral matches (databases and in-house data), isotopic distribution agreement, homologous-series patterns (including CF_2_-normalised Kendrick mass defects), characteristic PFAS fragments, and a literature comparison. In level 3, tentative candidates are selected, based on consistent retention times across replicates (±2.5%) and isotopic pattern fits >90%. Features containing fluorine and accurate mass errors within ±5 ppm were assigned to confidence level 4. Features were assigned to confidence level 5 when after peak alignment, grouping across samples, elemental composition prediction, background subtraction using mobile-phase blanks, showed an intensity threshold of 1.0 × 10^6^.

Each sample was injected three times for longer and shorter chain compounds. Following the application of predefined filters, data from both analyses were integrated. Then, only features containing fluorine were kept, and features not detected in at least two of the three injections were directly discarded. As a result, 79 features were kept at confidence level 4, 45 suspects were classified at level 3 (6 were discarded because they were not PFAS) so left a total of 39; from those, 31 suspects were tentatively identified at confidence level 2, and confirmation for 17 suspects was only established for the compounds that were confirmed by standards. Table 2 presents the tentatively identified compounds at confidence levels 3 and 2, and those confirmed and quantified at level 1.

It is noteworthy that there was the presence of short and ultrashort chain compounds and different fluorotelomer carboxylic acids, such as the 1:3 fluorotelomer carboxylic acid in 59% of the samples and 1:2 fluorotelomer carboxylic acid in 45%. These compounds were tentatively identified at levels 2 and 3, and they can be an indication of other sources of exposure, such as the ingestion of contaminated dust, which has been shown to be a relevant source of exposure to polyfluoroalkyl phosphate esters (PAPs) and other PFAS in household dust [49]. However, this hypothesis should be confirmed by the analysis of real dust where the child is exposed, which was not available at the time of this work. The main origin of fluorotelomer carboxylic acids is the degradation in the environment, or as metabolites [50] of fluorotelomer alcohols, which are used in a variety of consumer products as surfactants in food-contact materials and personal care products [51]. Another relevant compound found at tentative identification level 2 in 53% of the urine samples was N-methylperfluoro-1-octanesulfonamidoacetic acid (N-MePFOSAA), which is known to be a metabolite of PFOS.

The suspect screening approach enabled the tentative identification of several non-target PFAS, including fluorotelomer carboxylic acids, other fluorotelomers, and metabolites such as N-MePFOSAA, underlining the need for broader environmental and human monitoring. This is particularly important to assess the exposure of children to PFAS. The associations between PFAS exposure and adverse outcomes in children have been reported [52], including for respiratory and skin effects [53], pubertal development and effects on the thyroid hormones [54], neurotoxic effects [55], immune suppression [56], and increasing adiposity or cardiometabolic traits [57,58]. Recently, negative effects on psychological development have been a major concern. However, given the vast number of PFAS and their transformation products, and the diverse targeted analyses that pre-select a limited number of compounds, it is challenging to compare findings across studies, leading to mixed and sometimes contradictory results.

However, nowadays, it seems that evidence indicates that PFAS mixtures are overall positively associated with behavioural problems in children [59]. For example, the CHildhood Autism Risks from Genetics and Environment (CHARGE) case–control study related the exposure to per- and polyfluoroalkyl substances to increased odds of autism spectrum disorder in childhood [60]. In another longitudinal cohort study, the Tracing the Environmental Determinants of the Development of Your Child (TEDDY) child study found positive associations between PFAS exposure and behavioural issues among school-age children [61]. Also, PFAS mixtures were overall positively associated with behavioural problems among Chinese preschoolers [62]. Recently, the relationship between PFAS and attention-deficit/hyperactivity disorder has also been presented [18]. Therefore, it is of particular importance to have available methods that can provide a more complete picture of the different PFAS that can contribute to common effects. In particular, evidence suggests that restrictions on legacy PFAS have altered pollution profiles, and that concentrations of emerging, short and ultrashort chain compounds may surpass those of conventional PFAS in some contexts and their toxicity, persistence, and bioaccumulation potential, raise significant concerns regarding their human health impacts, as happens with perfluoroalkyl phosphinic acids [22].

## 3. Methods and Materials

### 3.1. Study Population and Recruitment

A summary of the participants’ diet and characteristics (*n* = 281) is given in Table S1 of the Supplementary Materials. Participants ranged in age from 7 to 8 years (mean 7.9 years), with 55% males and 45% females. Fifty-four percent of participants had an average body mass index (BMI) (kg/m^2^) within the normal range, while 16% were overweight, 24% were obese, and 6% were below the average weight.

The population in this study is part of the INMA Asturias birth cohort, established in 2004 by the University of Oviedo at San Agustin Hospital (Avilés, Asturias, North-West Spain) as part of the INMA [*Infancia y Medio Ambiente* (Environment and Childhood)] project [63]. The INMA Project aims to assess children’s exposure to environmental pollutants from pregnancy through childhood and evaluate their effects on growth, health, and development. It also investigates how genetic and nutritional factors may influence these effects. The eligibility criteria, as well as details on participant withdrawal and loss to follow-up, have been previously described [64,65]. Questionnaires on anthropometric and sociodemographic characteristics and lifestyle variables were performed in person with the assistance of trained personnel. All participants resided in the same geographic area.

During the 7–8-year follow-up period (2011–2016), urine samples were collected and stored at −20 °C, c.a. eight years prior to analysis. Due to the high stability of PFA, degradation when stored at −20 °C has been proven to be negligible. For the present analysis, PFAS were measured in 281 urine samples from participants aged 8 years who had available dietary information from a semiquantitative food frequency questionnaire (SQFFQ). This age was selected because it represents a key developmental stage: children are in middle childhood, a period of active neurodevelopment, and some, particularly girls, may begin showing early signs of puberty. PFAS exposure has been linked to endocrine and neurodevelopmental outcomes, making this an important age for biomonitoring. Parents also completed a questionnaire providing the demographic and dietary data reported in Table S1 of the Supplementary Materials.

The study protocol was approved by the Asturias Regional Ethics Committee, and written informed consent was obtained from parents or legal guardians from each participating child. The sample collection and research conformed to the principles of the Declaration of Helsinki.

Finally, the present study is an observational birth cohort study involving participants who all reside in the same geographic area, following the systematic analysis of cohort studies to analyse association and effects of covariates. In this context, no separate control group was included, since the research design aimed to describe PFAS occurrence within this population rather than compare exposure between distinct groups.

### 3.2. Chemical and Reagents

The list of abbreviations is presented in List S1 of the Supplementary Materials. Pure standards used for method validation and quantification included: (i) a PFAS mixture (MHX; >98%, Wellington Laboratories, Guelph, ON, Canada) in methanol containing carboxylates—PFBA, PFPeA, PFHxA, PFHpA, PFOA, PFNA, PFDA, PFUdA, PFDoA, PFTrDA, PFTeDA, PFHxDA, PFODA; sulfonates (PFBS, PFPeS, PFHxS, PFHpS, PFOS, PFNS, PFDS, PFDoS); and fluorotelomer sulfonates (4:2 FTS, 6:2 FTS, and 8:2 FTS); (ii) 6:2 diPAP, 8:2 diPAP, GenX (HFPO-DA), and ADONA, also from Wellington Laboratories; and (iii) trifluoroacetic acid (TFA), purchased from Sigma-Aldrich (Munich, Germany).

For instrumental signal normalisation, a mixture of isotopically labelled PFAS (MPFAC-HIF-ES; >98%, Wellington Laboratories) and a 13C2-TFA standard (Toronto Research Chemicals, Toronto, ON, Canada) was added prior to extraction (see Table S2 of the SM for details).

HPLC-grade methanol and water (CHROMASOLV^®^ Plus), ammonium acetate (≥98%), ammonium hydroxide (≥98%), and formic acid were obtained from Sigma-Aldrich (Steinheim, Germany). HPLC-grade acetonitrile (ACN) was purchased from Merck.

### 3.3. Sample Pretreatment

Urine samples were collected at home and stored in a clean polypropylene plastic container (100 mL), transported in cool conditions the same day, and frozen at −20 °C before analysis.

Sample pre-treatment was based on a previously published protocol that is shown in Figure S2 of the Supplementary Materials (SM) [66]. Briefly, 200 µL of urine was dispensed into an Eppendorf tube, to which 5 µL of a mixture of internal standards was added with the 27 selected compounds (Table S2 of SI), which allowed for the quantification of 29 compounds. The mixture was allowed to rest for 20 min for the purpose of equilibrium. Then, to precipitate traces of proteins, 200 µL of ACN was added, and the mixture was shaken for 5 min. The samples were centrifuged at 4000 rpm at 17 °C for 10 min. Finally, without disturbing the precipitate, 200 µL of supernatant was collected and introduced into a liquid chromatography vial with an insert.

### 3.4. Instrumental Analysis

PFAS analysis was performed using ultra-high performance liquid chromatography (UHPLC; Acquity, Waters Corporation, Milford, MA, USA) coupled to a Q-Exactive HRMS system (Thermo Fisher Scientific, San Jose, CA, USA) equipped with an electrospray ionisation (ESI) source operating in negative mode.

Short-chain PFAS separation was achieved using a mixed-mode Atlantis™ Premier BEH C18 AX column (2.1 × 100 mm, 2.5 µm; Waters), which provides enhanced retention and resolution for ultrashort- and short-chain PFAS compared with standard C18 columns [67]. The column was maintained at 40 °C, and the mobile phases consisted of 10 mM ammonium formate in water (A) and methanol (B). The gradient began at 5% B (1 min), increased to 45% over 4 min, then to 95% over another 4 min, was held for 2 min, and re-equilibrated at 5% for 3 min (0.3 mL/min). Long-chain PFAS were separated using a Hypersil GOLD PFP C18 column (50 × 3 µm; Thermo Fisher Scientific), selected for its high chromatographic resolution and capacity to minimise matrix interferences [66]. Mobile phases consisted of (A) methanol with 20 mM ammonium acetate and (B) water with 20 mM ammonium acetate. The gradient started at 10% A (1 min), increased to 90% over 10 min, was maintained for 2 min, and returned to 10% within 1 min (0.5 mL/min). Total run time for both methods was 12 min, with a 10 µL injection volume.

The UHPLC system was coupled to a Q-Exactive Orbitrap mass spectrometer using an electrospray ionisation (ESI) source for short-chain PFAS and heated electrospray ionisation (HESI) for long-chain PFAS, both under negative ionisation. For short-chain PFAS, MS parameters were nitrogen temperature of 350 °C, nebuliser of 40 psi, sheath gas temperature of 350 °C, sheath gas of 10 L/min, voltage of 350 V, capillary voltage of 3000 V, and nozzle of 1000 V (MS/MS range 100–1500 *m*/*z*). For long-chain PFAS: nitrogen at 350 °C, nebuliser of 50 psi, sheath gas temperature of 350 °C, sheath gas of 10 L/min, voltage of 250 V, capillary voltage of 2500 V, and nozzle of 1000 V (MS/MS range 60–900 *m*/*z*). Full-scan MS and MS/MS spectra were acquired using DIA/AIF modes to maximise the detection of fragmented ions.

### 3.5. Data Processing and Analysis

Data processed in Xcalibur Qual Browser (Thermo Fisher Scientific, San Jose, CA, USA) were further evaluated using Compound Discoverer 3.3 SP2. A customised PFAS database was compiled incorporating entries from external resources, including the NIST PFAS database [68], ChemSpider for structural information, and MzCloud for MS spectral data. The resulting database included structural descriptors, monoisotopic masses, and physicochemical properties (e.g., log P, log D) and supported the suspect-screening workflow following previously described criteria for mobile compounds.

The identification workflow was adapted from Schymanski et al. [48] (Figure S3). Confidence level 5 included peak alignment, unknown feature detection, grouping across samples, elemental composition prediction, background subtraction using mobile-phase blanks, and an intensity threshold of 1.0 × 10^6^. The 1.0 × 10^6^ intensity threshold was chosen to ensure robust AIF fragment deconvolution and minimise false positives, but it may indeed underestimate low-abundance PFAS, an inherent trade-off in AIF-based suspect screening. This limitation is acknowledged, and the combined targeted + suspect-screening approach compensates for reduced sensitivity in the AIF domain. Features containing fluorine and accurate mass errors within ±5 ppm were assigned to confidence level 4. From these, features showing consistent retention times across replicates (±2.5%) and isotopic pattern fits >90% were upgraded to level 3. Because AIF acquisition was used, enabling full MS/MS fragmentation without precursor selection, sufficient information was available for further filtration. AIF deconvolution avoided chimeric fragments by matching fragment ions to precursor ions through retention–time correlation, intensity co-elution, accurate-mass and isotopic filters, blank subtraction, and PFAS-specific structural constraints. This allowed for the reconstruction of clean, compound-specific MS/MS spectra even without precursor isolation. However, while the MS/MS spectra are deconvoluted to a degree, they still cannot be directly linked to a specific compound due to the DIA mode, which is a limitation. Tentative level 2 identifications were achieved through MS^2^ spectral matches (databases and in-house data), isotopic distribution agreement, homologous-series patterns (including CF_2_-normalised Kendrick mass defects), characteristic PFAS fragments, and literature comparison. Final confirmation (level 1) required matching with analytical standards.

### 3.6. Statistical Analysis

Data treatment and multivariate analysis were undertaken with GraphPad Prism version 10.1.1 (270). The data analysis was performed using the open-source software R with RStudio 2023.12.1+402, using the package “pheatmap” and hierarchical clustering with Ward’s D as the clustering method and Euclidean distance.

For comparison purposes, each tentatively identified compound was normalised to creatinine levels to account for hydration, and to its highest peak area among all samples in which it was identified. Therefore, the final datasheet used for statistical analysis comprises values from 0 to 1.

### 3.7. QA/QC and Validation Parameters

Method validation and calibration were performed using non-certified reference urine (non-CRM) from a 6-year-old donor containing residual short-chain PFAS. Five millilitres of this non-CRM were extracted following the procedure described in Section 3.3 and used for calibration and validation.

Calibration curves were prepared by spiking extracted non-CRM with a mixed standard solution at 500 ng/mL and diluting to obtain calibration points ranging from 0.001 to 500 ng/mL in the same extracted matrix.

Linearity, limits of detection (LODs), limits of quantification (LOQs), and precision were assessed following the SANTE/12682/2019 guidelines [69]. Four calibration points were injected in triplicate, and linearity was evaluated across 0.001–500 ng/mL for native standards. The method limit of detection (MLOD) was determined according to EPA guidance [70] using the lowest spiking level (*n* = 6), yielding values between 0.002 and 0.7 ng/mL. The method limit of quantification (MLOQ), defined as the lowest concentration meeting full method criteria with acceptable precision, ranged from 0.006 to 0.6 ng/mL; analyte-specific values are provided in Table S3 of the SM. In this table, the validation of 21 compounds parameters in urine including the 17 compounds quantified in the samples are presented. Precision was assessed through spiking at 0.001, 0.005, and 0.010 ng/mL, with inter-day precision expressed as the relative standard deviation and trueness derived from mean recoveries. Matrix effects were evaluated by comparing responses in matrix-matched standards (non-CRM) and neat solutions following Commission Decision 2002/657/CE. Quantification employed an internal standard for targeted congeners detected in the samples.

All laboratory consumables (e.g., vials, filters) were pre-screened for PFAS contamination. To minimise background levels, LC solvent lines were replaced with metallised or PEEK tubing, PTFE frits were substituted with stainless-steel frits, and polypropylene or glass containers were used to avoid PFAS leaching. Sample and laboratory blanks were included in each batch, and negative controls (blank samples/HPLC water) were analysed at 10% of the total sample count. Blank contamination remained below 5% of the sample signal. All samples were analysed in triplicate, and the mean and median concentrations were reported.

## 4. Conclusions

This study provides one of the first comprehensive assessments of legacy and emerging PFAS in children’s urine from an industrialised region of northwest Spain, addressing the lack of biomonitoring data for this vulnerable population. We found that emerging and highly mobile PFAS, particularly TFA (63%) and GenX (27%), dominated children’s exposure, and TFMS and ADONA were detected at lower frequencies of 3.2% and 1.4%, respectively, whereas legacy PFAS occurred infrequently and at low concentrations. Perfluorocarboxylic acids were more prevalent than sulfonates, consistent with their faster renal elimination, while the detection of TFMS, TFA, and PFBS highlights the presence of persistent, mobile, and understudied PFAS likely linked to the water cycle.

Although dietary items and dust could not be analysed directly, associations with previously reported food concentrations and the participants’ dietary patterns suggest contributions from dairy products, protein-rich foods, vegetables, and drinking water, in agreement with the EFSA estimates for European children. Future work, including real dietary and environmental samples, is needed to confirm exposure sources experimentally.

When examining potential differences between sexes, no significant differences were observed overall, except for PFBS (*p* = 0.000937), PFPeA (*p* = 0.000956), PFNA (*p* = 0.001338), and PFHpA (*p* = 0.002269), which showed statistically significant differences. The combined targeted and suspect-screening approach revealed additional PFAS, including previously undetected fluorotelomer carboxylic acids (e.g., 1:2 and 1:3 FTCA), demonstrating the value of LC-HRMS screening for capturing novel or unregulated PFAS and underscoring the need for expanded monitoring. These findings support regulatory discussions on the inclusion of emerging PFAS in food, water, and consumer-product safety frameworks, especially because some short-chain PFAS may still pose health risks following long-term exposure.

Finally, this study shows that urine is an effective biomatrix for assessing exposure to short- and ultrashort-chain PFAS, though it is suspected to be less suitable for long-chain congeners due to limited renal clearance based on the current literature. Overall, the findings emphasise the need for strengthened environmental regulation and continued research into the health implications of replacement, short-, and ultrashort-chain PFAS, particularly in children.

## Figures and Tables

**Table 1:** Summary of PFAS detected in urine samples of 281 children from the Asturias cohort (northwest Spain). Maximum (MAX) levels are in ng/mL; Minimum concentration (MIN) is in ng/mL, mean value and median are also in ng/mL.

	Frequency (%)	Results in Urine				Creatinine Normalised Values			
		MAX (ng/mL)	MIN (ng/mL)	Mean (ng/mL)	Median (ng/mL)	MAX (ng/mL)	MIN (ng/mL)	Mean (ng/mL)	Median (ng/mL)
**TFA**	**63**	**6.397**	0.180	1.553	0.972	**7.561**	0.212	1.836	1.148
**TFMS**	**3.2**	0.282	0.097	0.208	0.209	0.333	0.115	0.246	0.247
**ADONA**	1.4	0.051	0.023	0.037	0.037	0.060	0.027	0.044	0.044
**GenX**	**27**	**8.572**	0.244	1.338	1.066	**10.132**	0.288	1.581	1.261
**PFBA**	15.6	3.236	0.033	0.820	0.555	3.825	0.039	0.970	0.656
**PFPeA**	12.1	3.320	0.018	0.967	0.761	3.924	0.015	1.110	0.887
**PFHxA**	26	2.893	0.038	0.457	0.238	3.420	0.045	0.540	0.282
**PFHpA**	13.1	1.414	0.220	0.766	0.650	1.671	0.260	0.905	0.768
**PFOA**	**8.5**	**0.562**	0.063	0.292	0.306	**0.664**	0.074	0.345	0.363
**PFNA**	9.2	0.962	0.033	0.229	0.107	1.137	0.039	0.271	0.126
**PFDA**	2.1	0.497	0.169	0.267	0.233	0.587	0.199	0.315	0.275
**PFUnDA**	4.6	1.222	0.082	0.319	0.114	1.444	0.097	0.377	0.135
**PFBS**	15.5	3.274	0.320	1.381	1.200	3.870	0.378	1.633	1.418
**PFPeS**	5	0.174	0.015	0.065	0.047	0.206	0.018	0.073	0.053
**PFHxS**	5.3	1.761	0.211	0.529	0.361	2.081	0.249	0.625	0.427
**PFHpS**	3.2	0.870	0.110	0.266	0.160	1.028	0.130	0.314	0.189
PFOS *	**3.2**	**0.680**	0.147	0.384	0.465	**0.804**	0.174	0.498	0.570

Bolds included as a referee request. TFMS (trifluoromethane sulfonic acid); TFA (trifluoroacetic acid); ADONA (4.8-dioxa-3H perfluorononanoic acid); HFPO-DA or GenX (hexafluoropropylene oxide dimer acid); PFBA (perfluorobutanoic acid); PFPeA (perfluoropentanoic acid); PFHxA (perfluorohexanoic acid); PFHpA pPerfluoroheptanoic acid); PFOA (perfluorooctanoic acid); PFNA (perfluorononanoic acid); PFDA (perfluorodecanoic acid); PFUnDA (perfluoroundecanoic acid); PFBS (perfluobutanesulfonic acid); PFPeS (perfluoropentane sulfonic acid); PFHxS (perfluorohexane sulfonic acid); PFHpS (perfluoroheptanesulfonic acid); PFOS (perfluorooctane sulfonic acid), * MIN are the minimum quantities over the method limit of quantification (MLOQ).

**Table 2:** List of PFAS detected at confidence identification levels 1, 2, and 3, and the detection frequency in each case.

Name	Formula	Confidence Level	Frequency (%)	RT (min)	Exact Mass	Error (Δppm)	Database
Trifluoromethane sulfonic acid (TFMS)	CHF_3_O_3_S	1	3.2	2.94	149.9598	0.05	In-house
Trifluoro carboxylic acid (TFA)	C_2_HF_3_O_2_	1	63.1	1.83	113.9928	1.60	In-house
1:2 Fluorotelomer acid	C_3_H_3_F_3_O_2_	3	45	0.81	128.0085	2.18	PFAS NIST
N-Ethyl perfluoromethane sulfonamide	C_3_H_6_F_3_NO_2_S	3	4.3	0.50	177.0071	5.01	PFAS NIST
Perfluorobutanoic acid (PFBA)	C_4_HF_7_O_2_	1	15.6	6.45	213.9865	0.38	In-house
Perfluorobutanesulfonic acid (PFBS)	C_4_HF_9_O_3_S	1	15.5	7.28	299.9503	−0.81	In-house
1:3 Fluorotelomer carboxylic acid	C_4_H_5_F_3_O_2_	2	59	2.10	142.0242	−2.45	PFAS NIST
2:2 Unsaturated fluorotelomer carboxylic acid	C_4_H_2_F_4_O_2_	2	9.2	1.33	157.9991	−5.42	PFAS NIST
Vinyl trifluoroacetate	C_4_H_3_F_3_O_2_	3	6.3	0.60	140.0085	3.64	PFAS NIST
Perfluoropentanoic acid (PFPeA)	C_5_HF_9_O_2_	1	12.1	7.35	263.9833	−1.01	In-house
Perfluoropentane sulfonic acid (PFPeS)	C_5_HF_11_O_3_S	1	5.0	7.87	349.9471	−0.83	In-house
2,2,3,3,4,4,4-Heptafluoro-1-methoxybutan-1-ol	C_5_H_5_F_7_O_2_	2	10	0.57	230.0178	2.5	PFAS NIST
Perfluorohexanoic acid (PFHxA)	C_6_HF_11_O_2_	1	26	8.00	313.9801	0.57	In-house
Perfluorohexane sulfonic acid (PFHxS)	C_6_HF_13_O_3_S	1	5.3	8.35	399.9439	−0.96	In-house
HFPO-DA (GenX)	C_6_HF_11_O_3_	1	27.0	8.11	329.9750	0.22	In-house
Perfluoroheptanoic acid (PFHpA)	C_7_HF_13_O_2_	1	13.1	8.53	363.9769	0.06	In-house
4.8-Dioxa-3H perfluorononanoic acid (ADONA)	C_7_H_2_F_12_O_4_	1	1.4	8.51	377.9761	−1.04	In-house
Perfluorooctanoic acid (PFOA)	C_8_HF_15_O_2_	1	8.5	8.95	413.9737	−0.57	In-house
Perfluorooctane sulfonic acid (PFOS)	C_8_HF_17_O_3_S	1	3.2	9.11	499.9375	−0.39	In-house
3-Fluorophthalic acid	C_8_H_5_FO_4_	3	4.8	1.24	184.0172	−5.27	PFAS NIST
Perfluorononanoic acid (PFNA)	C_9_HF_17_O_2_	1	9.2	9.31	463.9705	−0.35	In-house
Perfluorononanesulfonic acid (PFNS)	C_9_HF_19_O_3_S	1	1.1	9.40	549.9343	−1.37	In-house
1-(Trifluoromethyl)-2-vinylbenzene	C_9_H_7_F_3_	2	0.9	0.54	172.0500	−5.80	PFAS NIST
Perfluorodecanoic acid (PFDA)	C_10_H F_19_O_2_	1	2.1	9.61	513.9673	−0.99	In-house
Perfluoroundecanoic acid (PFUnDA)	C_11_HF_21_O_2_	1	4.6	9.87	563.9641	−2.85	In-house
Unsaturated fluoro diEther dodecanoic acid	C_10_HF_17_O_4_	2	29	9.97	507.9603	−4.80	ChemSpider
3:3 Fluorotelomer betaine	C_10_H_15_F_7_NO_2_	2	11	0.58	314.0991	−3.55	PFAS NIST
Perfluorodecylphosphonic acid	C_10_H_2_F_21_O_3_P	3	0.5	9.24	599.9406	5.17	PFAS NIST
3,4,5,5,6,6,7,7,8,8,9,9,10,10,10-Pentadecafluorodec-3-en-2-one	C_10_H_3_F_15_O	2	9.7	9.31	507.9639	−0.94	PFAS NIST
N-Methylperfluoroalkanesulfonamidoacetic acid	C_10_H_6_F_15_NO_4_S	2	4.8	7.45	520.9765	−3.18	ChemSpider
1H,1H,2H,2H-Perfluorodecylamine	C_10_H_6_F_17_N	3	2.9	5.87	463.0229	−4.59	PFAS NIST
N-Methylperfluoro-1-octanesulfonamidoacetic acid (N-MeFOSAA)	C_11_H_6_F_17_NO_4_S	2	53	7.91	570.9729	−3.01	PFAS NIST
(3,3,4,4,5,5,6,6,7,7,8,8,9,9,10,10,11,11,12,12,12-Henicosafluorododecyl) phosphonic acid	C_12_H_6_F_21_O_3_P	3	1.4	9.01	627.9719	−4.91	PFAS NIST
2-(N-Ethylperfluorooctanesulfonamido) acetic acid (N-EtFOSAA)	C_12_H_8_F_17_NO_4_S	3	12	0.70	584.9903	−1.68	PFAS NIST
2-Ethyl-4-(1,1,1,2,3,3,3-heptafluoropropan-2-yl)-3-(methylsulfanyl)benzoic acid	C_13_H_11_F_7_O_2_S	2	3.4	6.20	364.0368	5.28	PFAS NIST
Methyl perfluorododecanoate	C_13_H_3_F_23_O_2_	2	16	7.46	627.9730	−5.67	PFAS NIST
Methyl pentacosafluorotridecanoate	C_14_H_3_ F_25_O_2_	2	13	7.92	677.9706	−4.00	PFAS NIST
12:2 fluorotelomer acid	C_14_H_3_F_25_O	2	3.4	7.74	661.9785	−8.19	PFAS NIST
12:2 Fluorotelomer sulfonamido propyl methyl amine	C_18_H_15_F_25_N_2_O_2_S	2	2.9	8.05	798.0455	9.34	PFAS NIST

## Data Availability

The original contributions presented in this study are included in the article/Supplementary Materials. Further inquiries can be directed to the corresponding authors.

## Supplementary Materials

The following supporting information can be downloaded at: https://www.mdpi.com/article/10.3390/molecules31050900/s1. List S1. List of Abbreviations. Figure S1. Extract ion chromatograms (XIC) with the exact masses of *m*/*z* selected PFASs. with an error within ±0.5 ppm. Figure S2. Schematic flowchart of the analytical method. Figure S3. Workflow for tentative identification at different confidence levels during suspect screening data treatment. Table S1. PFAS concentrations in urine samples. Table S2. List of Internal standards used as surrogate standards. Table S3. Quality assurance and quality control parameters of the analytical method.
