# Supplementary material for: Per- and Polyfluoroalkyl Substances in Urine Samples from Eight-Year-Old Children Living in Northwest Spain
Source: Molecules. 2026 Mar 9;31(5):900. doi: 10.3390/molecules31050900 (PMC12986414; doi:10.3390/molecules31050900)
Supplement: Supplementary file 1 [file molecules-31-00900-s001.zip › molecules-4107081-supplementary.pdf]

SUPPORTING INFORMATION

Per- and polyfluoroalkyl substances in urine samples from eight-year-old children living in northwest Spain.

Arianna Bautista [1]. Guillermo Fernández-Tardón [2]. Marta M. Rodríguez-Suárez [2]. Adonina Tardón [2]. Natalia Bravo [3]. Mercè Garí [3]. Joan O. Grimalt [3]. Marta Llorca [1*]. Marinella Farré [1*]

[1] ON-HEALTH group. Institute of Environmental Assessment and Water Research (IDAEA-CSIC). Barcelona. Spain
[2] Health Research Institute of Asturias (ISPA) and University of Oviedo. Asturias Spain
[3] Geochemistry and Pollution Group. Institute of Environmental Assessment and Water Research (IDAEA-CSIC). Barcelona. Spain

List S1. List of Abbreviations

Figure S1. Extract ion chromatograms (XIC) with the exact masses of *m*/*z* selected PFASs. with an error within ±0.5 ppm.

Figure S2. Schematic flowchart of the analytical method

Figure S3. Workflow for tentative identification at different confidence levels during suspect screening data treatment.

Table S1 PFAS concentrations in urine samples.

Table S2 . List of Internal standards used as surrogate standards.

Table S3 Quality assurance and quality control parameters of the analytical method.

SL1- List of Abbreviations

PFAS – Per- and polyfluoroalkyl substances
MHX – PFAS mixture from Wellington Laboratories
TFA – Trifluoroacetic acid
PFBA – Perfluorobutanoic acid
PFPeA – Perfluoropentanoic acid
PFHxA – Perfluorohexanoic acid
PFHpA – Perfluoroheptanoic acid
PFOA – Perfluorooctanoic acid
PFNA – Perfluorononanoic acid
PFDA – Perfluorodecanoic acid
PFUdA – Perfluoroundecanoic acid
PFDoA – Perfluorododecanoic acid
PFTrDA – Perfluorotridecanoic acid
PFTeDA – Perfluorotetradecanoic acid
PFHxDA – Perfluorohexadecanoic acid
PFODA – Perfluorooctadecanoic acid
PFBS – Perfluorobutanesulfonate
PFPeS – Perfluoropentanesulfonate
PFHxS – Perfluorohexanesulfonate
PFHpS – Perfluoroheptanesulfonate
PFOS – Perfluorooctanesulfonate
PFNS – Perfluorononesulfonate
PFDS – Perfluorodecanesulfonate
PFDoS – Perfluorododecanesulfonate

13C2-TFA – Mass-labelled trifluoroacetic acid
4:2 FTS – 1H.1H.2H.2H-Perfluorohexane sulfonate
6:2 FTS – 1H.1H.2H.2H-Perfluorooctane sulfonic acid
8:2 FTS – 1H.1H.2H.2H-Perfluorodecane sulfonate
6:2 diPAP – Sodium bis(1H.1H.2H.2H-perfluorooctyl)phosphate
8:2 diPAP – Sodium bis(1H.1H.2H.2H-perfluorodecyl)phosphate
GenX (HFPO-DA) – 2.3.3.3-Tetrafluoro-2-(1.1.2.2.3.3.3-heptafluoropropoxy)propanoic acid
ADONA – Sodium dodecafluoro-3H-4.8-dioxanonanoate

MPFAC-HIF-ES – Mixture of labelled PFASs from Wellington Laboratories
non-CRM – Non-certified reference material

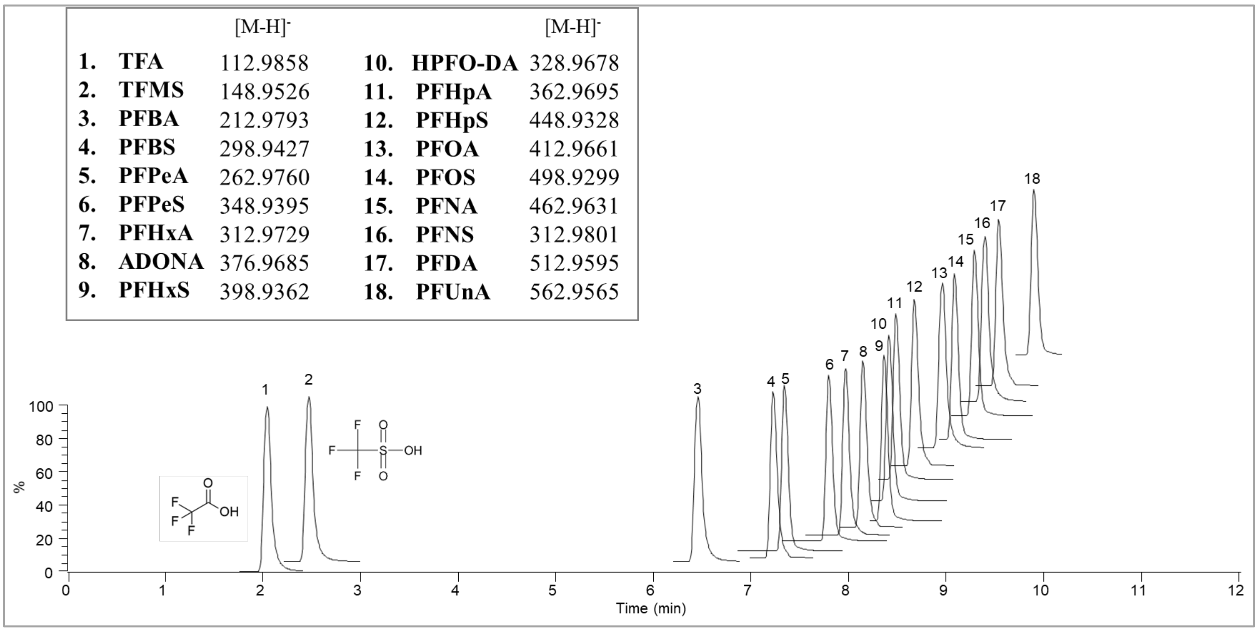

Figure S1. Extract ion chromatograms (XIC) with the exact masses of *m*/*z* selected PFASs. with an error within ±0.5 ppm.

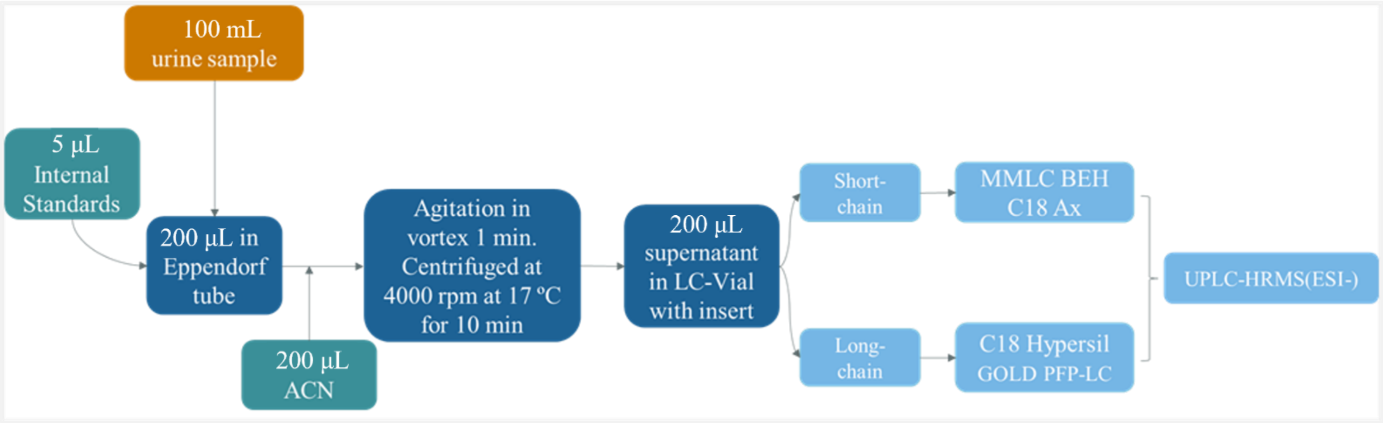

Figure S2. Schematic flowchart of the analytical method

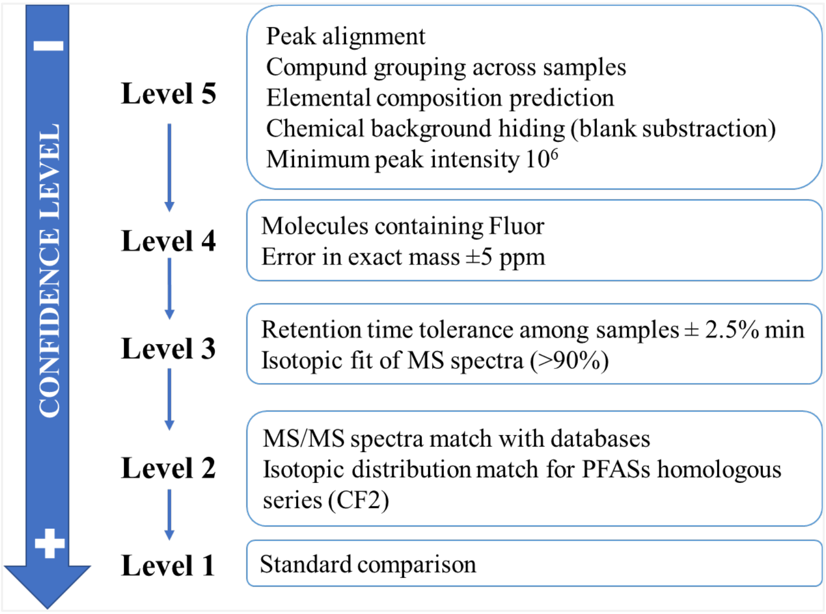

Figure S3. Workflow for tentative identification at different confidence levels during suspect screening data treatment.

Table S1. PFAS concentrations in urine samples

Samples	ng/mL															
	**TFA**	**TFMS**	**ADONA**	**GenX**	**PFBA**	**PFPeA**	**PFHxA**	**PFHpA**	**PFOA**	**PFNA**	**PFDA**	**PFUdA**	**PFBS**	**PFPeS**	**PFHxS**	**PFHpS**	**PFOS**
**1**	1.304	<MLOD	<MLOD	0.723	<MLOD	<MLOD	<MLOD	<MLOD	<MLOD	<MLOD	<MLOD	<MLOD	<MLOD	<MLOD	<MLOD	<MLOD	<MLOD
**7**	0.948	<MLOD	<MLOD	0.929	<MLOD	0.311	<MLOD	<MLOD	<MLOD	<MLOD	<MLOD	<MLOD	<MLOD	<MLOD	<MLOD	<MLOD	<MLOD
**10**	<MLOD	<MLOD	<MLOD	<MLOD	<MLOD	0.252	<MLOD	<MLOD	0.253	<MLOD	<MLOD	<MLOD	<MLOD	<MLOD	<MLOD	<MLOD	<MLOQ
**11**	4.545	<MLOD	0.023	<MLOD	<MLOQ	0.180	0.163	<MLOD	<MLOD	<MLOD	<MLOD	<MLOD	<MLOD	<MLOD	<MLOD	<MLOD	<MLOD
**12**	<MLOD	<MLOD	<MLOD	<MLOD	<MLOD	<MLOD	<MLOD	1.414	0.562	<MLOD	<MLOD	<MLOD	<MLOD	<MLOD	<MLOD	<MLOD	<MLOD
**15**	<MLOD	<MLOD	<MLOD	<MLOD	<MLOD	<MLOD	<MLOD	<MLOD	<MLOD	<MLOD	<MLOD	<MLOD	<MLOD	<MLOD	<MLOD	<MLOD	<MLOD
**18**	1.359	<MLOD	<MLOD	1.043	0.660	<MLOD	<MLOD	<MLOD	<MLOD	<MLOD	<MLOD	<MLOD	<MLOD	<MLOD	<MLOD	<MLOD	<MLOD
**20**	<MLOD	<MLOD	<MLOD	1.626	<MLOD	<MLOD	<MLOD	<MLOD	<MLOD	<MLOD	<MLOD	<MLOD	<MLOD	<MLOD	<MLOD	<MLOD	<MLOD
**22**	0.594	<MLOD	<MLOD	<MLOD	<MLOD	1.345	0.264	<MLOD	<MLOQ	<MLOD	<MLOD	<MLOD	<MLOD	<MLOD	<MLOD	<MLOD	<MLOD
**31**	1.393	<MLOD	<MLOD	<MLOD	<MLOD	<MLOD	<MLOD	<MLOD	<MLOD	<MLOD	<MLOD	<MLOD	<MLOD	<MLOD	<MLOD	<MLOD	<MLOD
**32**	0.956	<MLOD	<MLOD	<MLOD	<MLOD	<MLOD	<MLOD	<MLOD	<MLOD	<MLOD	<MLOD	<MLOD	1.801	0.053	<MLOD	<MLOD	<MLOD
**34**	0.293	<MLOD	<MLOD	<MLOD	<MLOD	<MLOD	<MLOD	<MLOD	<MLOD	<MLOD	<MLOD	<MLOD	<MLOD	<MLOD	<MLOD	<MLOD	<MLOD
**41**	2.403	<MLOD	<MLOD	<MLOD	<MLOD	1.738	1.082	0.455	<MLOQ	0.101	<MLOD	<MLOD	<MLOD	<MLOD	<MLOD	<MLOD	<MLOD
**43**	<MLOD	<MLOD	<MLOD	<MLOD	<MLOD	1.398	<MLOD	<MLOD	0.082	<MLOD	<MLOD	<MLOD	<MLOD	<MLOD	1.761	<MLOD	<MLOD
**45**	<MLOD	<MLOD	<MLOD	<MLOD	<MLOD	<MLOD	<MLOD	<MLOD	<MLOD	0.043	<MLOD	<MLOD	<MLOD	<MLOD	<MLOD	<MLOD	<MLOD
**47**	1.307	<MLOD	<MLOD	<MLOD	<MLOD	<MLOD	<MLOD	<MLOD	<MLOD	<MLOD	<MLOD	<MLOD	<MLOD	<MLOD	<MLOD	<MLOD	<MLOD
**56**	<MLOD	<MLOD	<MLOD	0.327	<MLOQ	1.200	<MLOD	1.314	0.341	<MLOD	<MLOD	<MLOD	<MLOD	<MLOD	0.631	<MLOD	<MLOD
**57**	<MLOD	<MLOD	<MLOD	<MLOD	0.450	<MLOD	<MLOD	<MLOD	<MLOD	<MLOD	<MLOD	<MLOD	<MLOD	<MLOD	<MLOD	<MLOD	<MLOD
**63**	<MLOD	<MLOD	<MLOD	<MLOD	<MLOD	1.300	0.123	<MLOD	<MLOD	0.054	<MLOD	<MLOD	0.341	<MLOD	0.563	<MLOD	<MLOD
**70**	0.254	<MLOD	<MLOD	<MLOD	<MLOD	<MLOD	0.345	<MLOD	0.283	<MLOD	<MLOD	<MLOD	0.657	<MLOD	<MLOD	<MLOD	<MLOD
**71**	1.489	<MLOD	<MLOD	<MLOD	<MLOD	<MLOD	0.137	0.411	<MLOQ	0.064	<MLOD	<MLOD	<MLOD	<MLOD	<MLOD	<MLOD	<MLOD

**80**	0.340	<MLOD	<MLOD	<MLOD	<MLOD	<MLOD	2.729	<MLOD	<MLOD	<MLOD	<MLOD	<MLOD	<MLOD	<MLOD	<MLOD	<MLOD	<MLOD
**82**	<MLOD	<MLOD	<MLOD	<MLOD	<MLOD	1.450	0.333	<MLOD	0.562	<MLOD	<MLOD	<MLOD	<MLOD	<MLOD	0.784	<MLOD	<MLOD
**85**	0.324	<MLOD	<MLOD	<MLOD	<MLOD	<MLOD	0.335	<MLOD	0.298	<MLOD	<MLOD	<MLOD	<MLOD	<MLOD	<MLOD	<MLOD	0.530
**89**	0.224	<MLOD	<MLOD	<MLOD	<MLOD	<MLOD	0.273	<MLOD	<MLOD	<MLOD	<MLOD	<MLOD	0.540	<MLOD	<MLOD	<MLOD	0.519
**94**	2.423	<MLOD	<MLOD	<MLOD	<MLOD	<MLOD	0.368	0.872	<MLOQ	0.173	<MLOD	<MLOD	<MLOD	<MLOD	<MLOD	<MLOD	<MLOD
**97**	0.321	<MLOD	<MLOD	2.062	0.051	<MLOD	<MLOD	<MLOD	<MLOD	<MLOD	<MLOD	<MLOD	<MLOD	<MLOD	<MLOD	<MLOD	<MLOD
**102**	0.595	<MLOD	<MLOD	<MLOD	<MLOD	<MLOD	<MLOD	<MLOD	<MLOD	<MLOD	<MLOD	<MLOD	<MLOD	<MLOD	<MLOD	<MLOD	<MLOD
**104**	0.504	<MLOD	<MLOD	<MLOD	<MLOD	<MLOD	<MLOD	<MLOD	<MLOD	<MLOD	<MLOD	<MLOD	2.090	<MLOD	<MLOD	<MLOD	<MLOD
**107**	1.111	<MLOD	<MLOD	<MLOD	<MLOD	<MLOD	<MLOD	<MLOD	<MLOD	<MLOD	<MLOD	<MLOD	0.320	<MLOD	<MLOD	<MLOD	<MLOD
**110**	0.263	<MLOD	<MLOD	<MLOD	<MLOD	<MLOD	<MLOD	<MLOD	<MLOD	<MLOD	<MLOD	<MLOD	<MLOD	<MLOD	<MLOD	<MLOD	<MLOD
**111**	1.855	<MLOD	<MLOD	<MLOD	<MLOD	<MLOD	0.166	0.577	<MLOD	0.084	<MLOD	<MLOD	<MLOD	<MLOD	<MLOD	<MLOD	<MLOD
**115**	<MLOD	<MLOD	<MLOD	<MLOD	<MLOD	<MLOD	<MLOD	<MLOD	<MLOD	<MLOD	<MLOD	<MLOD	<MLOD	<MLOD	<MLOD	<MLOD	<MLOD
**119**	1.933	<MLOD	<MLOD	<MLOD	<MLOD	<MLOD	0.162	<MLOD	<MLOD	<MLOD	<MLOD	<MLOD	<MLOD	<MLOD	<MLOD	<MLOD	<MLOQ
**125**	<MLOQ	<MLOD	<MLOD	<MLOD	<MLOD	<MLOD	<MLOD	<MLOD	<MLOD	<MLOD	<MLOD	<MLOD	<MLOD	<MLOD	<MLOD	<MLOD	<MLOD
**127**	0.217	<MLOD	<MLOD	<MLOD	<MLOD	<MLOD	0.148	<MLOD	0.371	0.433	<MLOD	<MLOD	<MLOD	<MLOD	0.541	<MLOD	0.447
**130**	0.370	<MLOD	<MLOD	<MLOQ	<MLOD	<MLOD	0.266	<MLOD	<MLOD	0.168	<MLOD	<MLOD	<MLOD	<MLOD	<MLOD	<MLOD	<MLOD
**136**	<MLOD	<MLOD	<MLOD	0.351	<MLOD	<MLOD	<MLOD	<MLOD	<MLOD	<MLOD	<MLOD	<MLOD	<MLOD	<MLOD	<MLOD	<MLOD	<MLOD
**144**	<MLOD	<MLOD	<MLOD	<MLOD	2.167	<MLOD	<MLOD	<MLOD	<MLOD	<MLOD	<MLOD	<MLOD	<MLOD	<MLOD	<MLOD	<MLOD	<MLOD
**148**	0.533	<MLOD	<MLOD	<MLOD	<MLOD	<MLOD	<MLOD	<MLOD	<MLOD	<MLOD	<MLOD	<MLOD	1.729	<MLOD	<MLOD	<MLOD	<MLOD
**152**	0.686	<MLOD	<MLOD	<MLOD	<MLOD	<MLOD	<MLOD	<MLOD	<MLOD	<MLOD	<MLOD	<MLOD	<MLOD	0.047	<MLOD	<MLOD	<MLOD
**155**	<MLOQ	<MLOD	<MLOD	0.638	<MLOD	<MLOD	<MLOD	<MLOD	<MLOD	<MLOD	<MLOD	<MLOD	<MLOD	<MLOD	<MLOD	<MLOD	<MLOD
**156**	0.446	<MLOD	<MLOD	<MLOD	<MLOD	<MLOD	<MLOD	0.220	<MLOD	0.414	0.299	<MLOD	<MLOD	0.174	0.281	<MLOD	<MLOD
**157**	<MLOD	<MLOD	<MLOD	<MLOD	<MLOD	1.652	<MLOQ	0.930	0.439	0.194	<MLOD	<MLOD	<MLOD	<MLOD	<MLOD	<MLOD	<MLOD
**159**	<MLOD	<MLOD	<MLOD	0.321	<MLOD	<MLOD	<MLOD	0.450	<MLOD	0.377	<MLOD	<MLOD	<MLOD	<MLOD	0.322	<MLOD	<MLOD

**162**	3.795	<MLOD	<MLOD	<MLOD	0.981	<MLOD	<MLOD	<MLOD	<MLOD	<MLOD	<MLOD	<MLOD	<MLOD	<MLOD	<MLOD	<MLOD	<MLOD
**174**	0.518	<MLOD	<MLOD	<MLOD	<MLOD	<MLOD	<MLOD	0.609	<MLOD	<MLOD	<MLOD	<MLOD	<MLOD	<MLOD	0.276	<MLOD	<MLOD
**180**	1.414	<MLOD	<MLOD	0.964	<MLOD	<MLOD	<MLOD	<MLOD	<MLOD	<MLOD	<MLOD	<MLOD	<MLOD	<MLOD	<MLOD	<MLOD	<MLOD
**200**	<MLOD	<MLOD	<MLOD	3.290	<MLOD	<MLOD	0.146	<MLOD	0.096	<MLOD	<MLOD	<MLOD	<MLOD	<MLOD	<MLOD	<MLOD	<MLOD
**203**	0.586	<MLOD	<MLOD	<MLOD	<MLOD	<MLOD	<MLOD	<MLOD	<MLOD	<MLOD	<MLOD	<MLOD	<MLOD	<MLOD	<MLOD	<MLOD	<MLOD
**210**	5.332	<MLOD	<MLOD	<MLOD	0.262	<MLOD	<MLOD	<MLOD	<MLOD	<MLOD	<MLOD	<MLOD	<MLOD	<MLOD	<MLOD	<MLOD	<MLOD
**214**	1.934	<MLOD	<MLOD	<MLOD	<MLOD	<MLOD	<MLOD	<MLOD	<MLOD	<MLOD	<MLOD	<MLOD	0.660	<MLOD	<MLOD	<MLOD	<MLOD
**217**	<MLOD	<MLOD	<MLOD	1.520	0.145	<MLOD	<MLOD	<MLOD	<MLOD	<MLOD	<MLOD	<MLOD	<MLOD	<MLOD	<MLOD	<MLOD	<MLOD
**218**	<MLOD	<MLOD	<MLOD	1.493	0.133	<MLOD	<MLOD	<MLOD	<MLOD	<MLOD	<MLOD	<MLOD	<MLOD	<MLOD	<MLOD	<MLOD	<MLOD
**224**	0.465	0.209	<MLOD	<MLOD	<MLOD	<MLOD	<MLOD	0.796	<MLOD	<MLOD	<MLOD	<MLOD	3.012	<MLOD	<MLOD	<MLOD	<MLOD
**225**	0.525	<MLOD	<MLOD	<MLOD	<MLOD	<MLOQ	<MLOD	1.190	<MLOD	<MLOD	<MLOD	<MLOD	2.142	0.079	<MLOD	<MLOD	<MLOD
**230**	<MLOD	<MLOD	<MLOD	1.491	<MLOD	<MLOD	<MLOD	<MLOD	<MLOD	<MLOD	<MLOD	<MLOD	<MLOD	<MLOD	<MLOD	<MLOD	<MLOD
**231**	0.226	<MLOD	<MLOD	<MLOD	<MLOD	<MLOD	0.327	<MLOD	<MLOD	<MLOD	<MLOD	<MLOD	<MLOD	<MLOD	<MLOD	<MLOD	<MLOD
**233**	<MLOD	<MLOD	<MLOD	<MLOD	<MLOD	<MLOD	<MLOD	<MLOD	<MLOD	<MLOD	<MLOD	<MLOD	<MLOD	<MLOD	0.604	<MLOD	<MLOD
**237**	0.795	<MLOD	<MLOD	<MLOD	<MLOD	<MLOD	<MLOD	0.604	<MLOD	<MLOD	<MLOD	<MLOD	1.347	<MLOD	<MLOD	<MLOD	<MLOD
**240**	<MLOD	<MLOD	<MLOD	3.046	2.165	<MLOD	<MLOD	0.650	<MLOD	<MLOD	<MLOD	<MLOD	0.451	<MLOD	<MLOD	<MLOD	<MLOD
**250**	0.391	<MLOD	<MLOD	<MLOD	<MLOD	0.340	1.715	0.670	<MLOD	0.231	<MLOD	<MLOD	<MLOD	<MLOD	0.761	<MLOD	<MLOD
**258**	1.498	<MLOD	<MLOD	<MLOD	<MLOD	1.452	<MLOD	0.780	<MLOD	0.452	<MLOD	<MLOD	<MLOD	<MLOD	<MLOD	<MLOD	<MLOD
**259**	0.707	<MLOD	<MLOD	<MLOD	<MLOD	<MLOD	<MLOD	1.219	<MLOD	<MLOD	<MLOD	<MLOD	<MLOD	<MLOD	<MLOD	0.183	<MLOD
**262**	1.903	<MLOD	<MLOD	<MLOD	<MLOD	<MLOQ	1.784	<MLOD	<MLOD	<MLOD	<MLOD	<MLOD	<MLOD	<MLOD	<MLOD	0.430	<MLOQ
**263**	1.373	<MLOD	<MLOD	<MLOD	<MLOD	3.320	<MLOQ	0.324	<MLOD	<MLOD	<MLOD	<MLOD	<MLOD	<MLOD	<MLOD	<MLOQ	<MLOD
**267**	0.468	<MLOD	<MLOD	<MLOD	<MLOD	<MLOD	<MLOD	1.235	<MLOD	<MLOD	<MLOD	<MLOD	<MLOD	0.062	<MLOD	<MLOD	<MLOD
**269**	0.195	<MLOD	<MLOD	<MLOD	<MLOD	<MLOD	<MLOD	<MLOD	<MLOD	<MLOD	<MLOD	<MLOD	0.622	<MLOD	<MLOD	<MLOD	<MLOD
**270**	0.560	<MLOD	<MLOD	<MLOD	<MLOD	<MLOD	<MLOD	<MLOD	<MLOD	<MLOD	<MLOD	<MLOD	2.061	0.161	<MLOD	<MLOD	<MLOD

**272**	1.445	<MLOD	<MLOD	<MLOD	<MLOD	<MLOD	<MLOD	<MLOD	<MLOD	<MLOD	<MLOD	<MLOD	<MLOD	<MLOD	<MLOD	<MLOD	<MLOD
**275**	<MLOD	<MLOD	<MLOD	<MLOD	<MLOD	<MLOD	<MLOD	<MLOD	<MLOD	<MLOD	<MLOD	<MLOD	<MLOD	<MLOD	<MLOD	<MLOD	<MLOD
**279**	<MLOD	<MLOD	<MLOD	1.644	0.354	0.654	<MLOD	<MLOD	<MLOD	<MLOD	<MLOD	<MLOD	<MLOD	<MLOD	<MLOD	<MLOD	<MLOD
**284**	<MLOD	<MLOD	<MLOD	<MLOD	<MLOD	<MLOD	<MLOD	<MLOD	<MLOD	<MLOD	<MLOD	<MLOD	<MLOD	<MLOD	<MLOD	<MLOD	<MLOD
**289**	2.638	<MLOD	<MLOD	<MLOD	<MLOD	<MLOD	<MLOD	<MLOD	<MLOD	<MLOD	<MLOD	<MLOD	<MLOD	<MLOD	<MLOD	<MLOD	<MLOD
**290**	4.286	<MLOD	<MLOD	<MLOD	<MLOD	<MLOD	0.205	<MLOD	<MLOD	0.104	<MLOD	<MLOD	<MLOD	<MLOD	<MLOD	<MLOD	<MLOD
**291**	0.898	<MLOD	<MLOD	<MLOD	<MLOD	<MLOD	<MLOD	1.168	<MLOD	<MLOD	<MLOD	<MLOD	<MLOD	0.043	<MLOD	0.087	<MLOD
**293**	<MLOD	<MLOD	<MLOD	8.572	<MLOD	<MLOD	<MLOD	<MLOD	<MLOD	<MLOD	<MLOD	<MLOD	<MLOD	<MLOD	<MLOD	<MLOD	<MLOD
**294**	<MLOD	<MLOD	<MLOD	<MLOD	<MLOD	0.244	0.038	<MLOQ	<MLOD	<MLOD	<MLOD	<MLOD	<MLOD	<MLOD	<MLOD	<MLOD	<MLOD
**299**	3.819	<MLOD	<MLOD	<MLOD	0.122	<MLOD	<MLOD	<MLOD	<MLOD	<MLOD	<MLOD	<MLOD	<MLOD	<MLOD	<MLOD	<MLOD	<MLOD
**304**	<MLOD	<MLOD	<MLOD	<MLOD	<MLOD	<MLOD	<MLOD	<MLOD	<MLOD	<MLOD	<MLOD	0.082	<MLOQ	<MLOD	<MLOD	<MLOD	<MLOD
**305**	<MLOD	<MLOD	<MLOD	2.776	<MLOD	<MLOD	0.282	<MLOD	0.315	<MLOD	<MLOD	<MLOD	<MLOD	<MLOD	<MLOD	<MLOD	<MLOD
**317**	2.468	<MLOD	<MLOD	<MLOD	<MLOD	<MLOD	<MLOD	<MLOD	<MLOD	0.108	<MLOD	<MLOD	<MLOD	<MLOD	<MLOD	<MLOD	<MLOD
**321**	<MLOQ	<MLOD	<MLOD	<MLOD	<MLOD	0.619	<MLOD	<MLOD	<MLOD	<MLOD	<MLOD	<MLOD	<MLOD	<MLOD	<MLOD	<MLOD	<MLOD
**326**	0.639	<MLOD	<MLOD	<MLOD	<MLOD	<MLOD	<MLOD	<MLOD	<MLOD	<MLOD	<MLOD	<MLOD	<MLOD	0.010	<MLOD	<MLOD	<MLOD
**330**	<MLOD	<MLOD	<MLOD	2.482	<MLOD	<MLOD	0.193	<MLOD	0.236	<MLOD	<MLOD	<MLOD	<MLOD	<MLOD	<MLOD	<MLOD	<MLOD
**339**	<MLOD	<MLOD	<MLOD	<MLOD	<MLOD	<MLOD	<MLOD	<MLOD	<MLOD	<MLOD	<MLOD	<MLOD	<MLOD	<MLOD	<MLOD	<MLOD	<MLOD
**341**	<MLOD	<MLOD	<MLOD	<MLOD	<MLOD	1.039	<MLOD	<MLOD	<MLOD	<MLOD	<MLOD	<MLOD	<MLOD	<MLOD	<MLOD	<MLOD	<MLOD
**343**	3.961	<MLOQ	0.051	<MLOD	<MLOD	0.284	0.188	<MLOD	<MLOD	0.105	<MLOD	<MLOD	<MLOD	<MLOD	<MLOD	<MLOD	<MLOD
**344**	<MLOD	<MLOD	<MLOD	1.067	<MLOD	<MLOD	<MLOD	<MLOD	<MLOD	<MLOD	<MLOD	<MLOD	<MLOD	<MLOD	<MLOD	<MLOQ	<MLOD
**347**	0.504	<MLOD	<MLOD	<MLOD	<MLOD	<MLOD	<MLOD	<MLOD	<MLOD	<MLOD	<MLOD	<MLOD	1.489	<MLOD	<MLOD	<MLOD	<MLOD
**348**	<MLOD	<MLOD	<MLOD	1.947	3.236	<MLOD	<MLOD	<MLOD	<MLOD	<MLOD	<MLOD	<MLOD	<MLOD	<MLOD	<MLOD	<MLOD	<MLOD
**349**	<MLOD	<MLOD	<MLOQ	0.244	<MLOD	<MLOD	<MLOD	<MLOD	<MLOD	<MLOD	<MLOD	<MLOD	<MLOD	<MLOD	<MLOD	<MLOD	<MLOD
**358**	<MLOD	<MLOD	<MLOD	<MLOD	<MLOD	<MLOD	0.849	1.318	<MLOQ	0.962	<MLOD	<MLOD	<MLOD	<MLOD	<MLOD	<MLOD	<MLOD

**362**	2.453	<MLOD	<MLOD	<MLOD	<MLOD	0.499	0.172	<MLOD	<MLOD	<MLOD	<MLOD	<MLOD	<MLOD	<MLOD	<MLOD	<MLOD	<MLOD
**363**	<MLOD	<MLOD	<MLOD	<MLOD	<MLOD	<MLOD	<MLOD	<MLOD	0.255	<MLOD	<MLOD	<MLOD	<MLOD	<MLOD	<MLOD	<MLOD	<MLOD
**374**	1.462	<MLOD	<MLOD	<MLOD	<MLOD	<MLOD	<MLOD	<MLOD	<MLOD	<MLOD	<MLOD	<MLOD	<MLOD	<MLOD	<MLOD	<MLOD	<MLOD
**377**	0.594	<MLOD	<MLOD	<MLOD	<MLOD	<MLOD	<MLOD	1.228	<MLOD	<MLOD	<MLOD	<MLOD	2.279	<MLOD	<MLOD	<MLOD	<MLOD
**378**	<MLOD	<MLOD	<MLOD	<MLOD	0.834	<MLOD	<MLOD	<MLOD	<MLOD	<MLOD	<MLOD	<MLOD	<MLOD	<MLOD	<MLOD	<MLOD	<MLOD
**381**	0.241	<MLOD	<MLOD	<MLOD	<MLOD	<MLOD	<MLOD	<MLOD	<MLOD	<MLOD	<MLOD	<MLOD	<MLOD	<MLOD	<MLOD	<MLOD	<MLOD
**384**	0.922	<MLOD	<MLOD	<MLOD	<MLOD	<MLOD	<MLOD	<MLOD	<MLOD	<MLOD	<MLOD	<MLOD	<MLOD	<MLOD	<MLOD	<MLOD	<MLOD
**401**	<MLOQ	<MLOD	<MLOD	<MLOD	<MLOD	<MLOD	<MLOD	<MLOD	<MLOD	<MLOD	<MLOD	<MLOD	0.554	<MLOD	<MLOD	<MLOD	<MLOD
**403**	0.190	0.237	<MLOD	<MLOD	<MLOD	0.761	0.367	1.060	<MLOD	<MLOD	<MLOD	<MLOD	0.618	<MLOD	<MLOD	<MLOD	<MLOD
**407**	0.576	<MLOD	<MLOD	<MLOD	<MLOD	<MLOD	<MLOD	<MLOD	<MLOD	<MLOD	<MLOD	<MLOD	<MLOD	<MLOD	<MLOD	<MLOD	<MLOD
**410**	4.409	<MLOD	<MLOD	0.748	<MLOQ	<MLOD	<MLOD	<MLOD	<MLOD	<MLOD	<MLOD	<MLOD	<MLOD	<MLOD	<MLOD	<MLOD	<MLOD
**413**	<MLOD	<MLOD	<MLOD	<MLOD	1.159	<MLOD	<MLOD	<MLOD	<MLOD	<MLOD	<MLOD	<MLOD	<MLOD	<MLOD	<MLOD	<MLOD	<MLOD
**415**	0.817	0.097	<MLOD	2.578	<MLOQ	0.894	2.893	<MLOD	<MLOD	<MLOD	<MLOD	<MLOD	2.909	<MLOD	<MLOD	0.110	<MLOD
**420**	0.530	<MLOD	<MLOD	<MLOD	<MLOD	<MLOD	<MLOD	<MLOD	<MLOD	<MLOD	<MLOD	<MLOD	<MLOD	<MLOD	<MLOD	0.160	<MLOD
**429**	0.612	<MLOD	<MLOD	<MLOD	<MLOD	<MLOD	<MLOD	<MLOD	<MLOD	<MLOD	<MLOD	<MLOD	1.645	0.025	<MLOD	<MLOD	<MLOD
**431**	0.378	<MLOD	<MLOD	<MLOD	<MLOD	<MLOD	<MLOD	1.161	<MLOD	<MLOD	<MLOD	<MLOD	1.052	<MLOD	<MLOD	0.127	<MLOD
**434**	1.570	<MLOD	<MLOD	0.776	<MLOD	<MLOD	<MLOD	<MLOD	<MLOD	<MLOD	<MLOD	<MLOD	<MLOD	<MLOD	<MLOD	<MLOD	<MLOD
**440**	0.714	<MLOD	<MLOD	<MLOD	<MLOD	<MLOD	<MLOD	<MLOD	<MLOD	<MLOD	<MLOD	<MLOD	<MLOD	<MLOD	<MLOD	<MLOD	<MLOD
**443**	0.992	0.202	<MLOD	<MLOD	<MLOD	<MLOD	<MLOD	<MLOD	<MLOD	<MLOD	<MLOD	<MLOD	<MLOD	0.016	<MLOD	<MLOD	<MLOD
**448**	0.264	<MLOD	<MLOD	<MLOD	<MLOD	<MLOD	<MLOD	<MLOD	<MLOD	<MLOD	<MLOD	<MLOD	<MLOD	<MLOD	<MLOD	<MLOD	<MLOD
**455**	0.193	<MLOD	<MLOD	<MLOD	<MLOD	<MLOD	<MLOD	<MLOD	<MLOD	<MLOD	<MLOD	<MLOD	1.814	<MLOD	<MLOD	<MLOD	<MLOD
**456**	<MLOD	<MLOD	<MLOD	0.426	<MLOQ	<MLOD	<MLOD	<MLOD	<MLOD	<MLOD	<MLOD	<MLOD	<MLOD	<MLOD	<MLOD	<MLOD	<MLOD
**459**	<MLOD	<MLOD	<MLOD	<MLOD	<MLOD	<MLOD	<MLOD	<MLOD	<MLOD	<MLOD	<MLOD	<MLOD	0.630	<MLOD	<MLOD	<MLOD	<MLOD
**460**	<MLOD	<MLOD	<MLOD	1.045	<MLOD	<MLOD	<MLOD	<MLOD	<MLOD	<MLOD	<MLOD	<MLOD	<MLOD	<MLOD	<MLOD	<MLOD	<MLOD

**461**	<MLOD	<MLOD	<MLOD	1.579	1.158	<MLOD	<MLOD	<MLOD	<MLOD	<MLOD	<MLOD	<MLOD	<MLOD	<MLOD	<MLOD	<MLOD	<MLOD
**463**	0.744	<MLOD	<MLOD	<MLOD	<MLOD	<MLOD	<MLOD	<MLOD	<MLOD	<MLOD	<MLOD	<MLOD	1.946	<MLOD	<MLOD	<MLOD	<MLOD
**464**	3.174	<MLOD	<MLOD	<MLOD	0.723	<MLOD	<MLOD	<MLOD	<MLOD	<MLOD	<MLOD	<MLOD	<MLOD	<MLOD	<MLOD	<MLOD	<MLOD
**467**	0.250	<MLOD	<MLOD	<MLOD	<MLOD	<MLOD	<MLOD	<MLOD	<MLOD	<MLOD	<MLOD	<MLOD	2.226	<MLOD	<MLOD	<MLOD	<MLOD
**470**	6.061	<MLOD	0.027	<MLOD	<MLOD	0.693	0.214	<MLOD	<MLOD	0.084	<MLOD	<MLOD	<MLOD	<MLOD	<MLOD	<MLOD	0.107
**471**	<MLOD	<MLOD	<MLOD	1.086	<MLOD	<MLOD	<MLOD	<MLOD	<MLOD	<MLOD	<MLOD	<MLOD	<MLOD	<MLOD	<MLOD	<MLOD	<MLOD
**481**	1.254	<MLOD	<MLOD	<MLOD	1.231	<MLOD	<MLOD	<MLOD	<MLOD	<MLOD	0.231	<MLOD	0.980	<MLOD	<MLOD	<MLOD	<MLOD
**483**	2.237	<MLOD	<MLOD	<MLOD	0.754	<MLOD	0.881	<MLOD	<MLOD	<MLOD	<MLOD	<MLOD	1.689	<MLOD	<MLOD	<MLOD	<MLOD
**484**	3.261	<MLOD	<MLOD	<MLOD	<MLOD	<MLOD	0.216	<MLOD	<MLOD	0.113	<MLOD	<MLOD	<MLOD	<MLOD	<MLOD	<MLOD	<MLOD
**488**	1.300	<MLOD	<MLOD	<MLOD	<MLOD	0.950	<MLOD	0.451	<MLOD	<MLOD	<MLOD	<MLOD	<MLOD	<MLOD	<MLOD	<MLOD	<MLOD
**500**	<MLOD	<MLOD	<MLOD	<MLOD	0.852	0.790	<MLOD	<MLOD	<MLOD	<MLOD	<MLOD	<MLOD	2.319	<MLOD	<MLOD	<MLOD	<MLOD
**507**	2.239	<MLOD	<MLOD	1.515	<MLOD	0.740	0.137	0.754	<MLOD	<MLOD	<MLOD	0.088	<MLOD	<MLOD	<MLOD	<MLOD	<MLOD
**511**	<MLOD	<MLOD	<MLOD	<MLOD	<MLOD	<MLOD	<MLOD	<MLOD	<MLOD	0.033	<MLOD	<MLOD	<MLOD	<MLOD	<MLOD	<MLOD	<MLOD
**515**	0.988	<MLOD	<MLOD	<MLOD	<MLOD	<MLOD	<MLOD	<MLOD	<MLOD	<MLOD	<MLOD	<MLOD	2.059	<MLOD	<MLOD	<MLOD	<MLOD
**520**	3.803	<MLOD	<MLOD	<MLOD	<MLOD	<MLOD	0.097	<MLOD	<MLOD	<MLOD	<MLOD	<MLOD	<MLOD	<MLOD	<MLOD	<MLOD	<MLOD
**526**	2.765	<MLOD	<MLOD	<MLOD	0.099	<MLOD	<MLOD	<MLOD	<MLOD	<MLOD	<MLOD	<MLOD	<MLOD	<MLOD	<MLOD	<MLOD	<MLOD
**532**	<MLOD	<MLOD	<MLOD	<MLOD	<MLOD	<MLOD	0.313	<MLOD	<MLOD	<MLOD	<MLOD	<MLOD	<MLOD	<MLOD	<MLOD	<MLOD	<MLOD
**536**	0.467	<MLOD	<MLOD	<MLOD	<MLOD	<MLOD	<MLOD	0.638	<MLOD	<MLOD	<MLOD	<MLOD	<MLOD	<MLOD	<MLOD	<MLOD	<MLOD
**537**	<MLOD	<MLOD	<MLOD	1.965	<MLOD	<MLOD	0.278	<MLOD	<MLOD	<MLOD	<MLOD	0.099	<MLOD	<MLOD	<MLOD	<MLOD	<MLOD
**538**	6.397	<MLOD	<MLOD	<MLOD	<MLOD	<MLOD	<MLOD	<MLOD	<MLOD	<MLOD	<MLOD	<MLOD	<MLOD	<MLOD	<MLOD	<MLOD	<MLOD
**540**	1.138	<MLOD	<MLOD	<MLOD	<MLOD	<MLOD	<MLOD	<MLOD	<MLOD	<MLOD	<MLOD	<MLOD	<MLOD	<MLOD	<MLOD	<MLOD	<MLOD
**542**	<MLOD	<MLOD	<MLOD	0.823	<MLOD	<MLOD	<MLOD	<MLOD	<MLOD	<MLOD	<MLOD	<MLOD	<MLOD	<MLOD	<MLOD	<MLOD	<MLOD
**543**	<MLOD	<MLOD	<MLOD	<MLOD	<MLOD	<MLOD	0.173	<MLOD	<MLOD	<MLOD	<MLOD	<MLOD	<MLOD	<MLOD	<MLOD	<MLOD	<MLOD
**546**	<MLOD	<MLOD	<MLOD	<MLOD	<MLOD	<MLOD	<MLOD	<MLOD	<MLOD	<MLOD	<MLOD	<MLOD	<MLOD	<MLOD	<MLOD	<MLOD	<MLOD

**547**	<MLOD	<MLOD	<MLOD	2.056	<MLOD	1.234	<MLOD	<MLOD	0.452	<MLOD	0.235	<MLOD	<MLOD	<MLOD	<MLOD	<MLOD	<MLOD
**550**	0.271	<MLOD	<MLOD	<MLOD	<MLOD	<MLOD	<MLOD	<MLOD	<MLOD	0.829	0.497	0.830	0.457	<MLOD	<MLOD	<MLOD	<MLOD
**552**	4.071	<MLOD	<MLOD	<MLOD	0.277	0.707	<MLOD	<MLOD	<MLOD	<MLOD	<MLOD	<MLOD	<MLOD	<MLOD	<MLOD	<MLOD	<MLOD
**553**	<MLOD	<MLOD	<MLOD	1.114	<MLOD	<MLOD	0.430	<MLOD	<MLOD	0.530	<MLOD	1.222	<MLOD	<MLOD	<MLOD	<MLOD	<MLOD
**556**	1.459	<MLOD	<MLOD	<MLOD	<MLOD	0.243	<MLOD	<MLOD	<MLOD	0.067	<MLOD	<MLOD	<MLOD	<MLOD	<MLOD	<MLOD	<MLOD
**559**	0.809	<MLOD	<MLOD	<MLOD	<MLOD	<MLOD	<MLOD	<MLOD	<MLOD	<MLOD	<MLOD	<MLOD	3.101	<MLOD	<MLOD	<MLOD	<MLOD
**563**	4.476	<MLOD	<MLOD	1.168	1.166	<MLOD	<MLOD	<MLOD	<MLOD	<MLOD	<MLOD	<MLOD	<MLOD	<MLOD	<MLOD	<MLOD	<MLOD
**565**	<MLOD	<MLOD	<MLOD	<MLOD	<MLOD	<MLOD	0.311	<MLOD	<MLOD	<MLOD	<MLOD	<MLOD	0.617	<MLOD	<MLOD	<MLOD	<MLOD
**569**	4.286	<MLOD	<MLOD	1.943	0.124	<MLOD	<MLOD	<MLOD	<MLOD	<MLOD	<MLOD	<MLOD	<MLOD	<MLOD	<MLOD	<MLOD	<MLOD
**570**	<MLOD	<MLOD	<MLOD	<MLOD	<MLOD	<MLOD	<MLOD	<MLOD	<MLOD	<MLOD	<MLOD	<MLOD	<MLOD	<MLOD	<MLOD	<MLOD	<MLOD
**572**	0.279	0.204	<MLOD	<MLOD	<MLOD	<MLOD	<MLOD	0.969	<MLOD	<MLOD	<MLOD	<MLOD	1.823	<MLOD	<MLOD	<MLOD	<MLOD
**573**	0.317	<MLOD	<MLOD	<MLOD	<MLOD	<MLOD	0.132	0.454	<MLOD	<MLOD	0.170	0.307	0.857	<MLOD	0.355	<MLOD	<MLOD
**575**	1.881	<MLOD	<MLOD	<MLOD	<MLOD	<MLOD	<MLOD	<MLOD	<MLOD	<MLOD	<MLOD	<MLOD	<MLOD	<MLOD	<MLOD	<MLOD	<MLOD
**578**	<MLOD	<MLOD	<MLOD	<MLOD	<MLOD	<MLOD	0.226	<MLOD	<MLOD	<MLOD	<MLOD	<MLOD	<MLOD	<MLOD	<MLOD	<MLOD	<MLOD
**581**	1.650	<MLOD	<MLOD	<MLOD	<MLOD	<MLOD	<MLOD	<MLOD	<MLOD	<MLOD	<MLOD	<MLOD	<MLOD	<MLOD	<MLOD	<MLOD	<MLOD
**585**	<MLOQ	<MLOD	<MLOD	0.284	0.033	<MLOD	<MLOD	<MLOD	<MLOD	<MLOD	<MLOD	<MLOD	<MLOD	<MLOD	<MLOD	<MLOD	<MLOD
**14**	4.242	<MLOD	<MLOD	<MLOD	<MLOD	<MLOD	<MLOD	<MLOD	<MLOD	<MLOD	<MLOD	<MLOD	<MLOD	<MLOD	<MLOD	<MLOD	<MLOD
**19**	<MLOD	<MLOD	<MLOD	<MLOD	0.218	<MLOD	<MLOD	<MLOD	<MLOD	<MLOD	<MLOD	<MLOD	<MLOD	<MLOD	<MLOD	<MLOD	<MLOD
**26**	0.808	<MLOD	<MLOD	<MLOD	<MLOD	0.296	<MLOD	<MLOD	<MLOD	<MLOD	<MLOD	<MLOD	<MLOD	<MLOD	<MLOD	<MLOD	<MLOD
**28**	0.441	<MLOD	<MLOD	<MLOD	<MLOD	<MLOD	<MLOD	<MLOD	<MLOD	<MLOD	<MLOD	<MLOD	<MLOD	<MLOD	<MLOD	<MLOD	<MLOD
**35**	<MLOD	<MLOD	<MLOD	<MLOD	<MLOD	<MLOD	0.312	<MLOD	0.422	<MLOD	<MLOD	<MLOD	<MLOD	<MLOD	<MLOD	<MLOD	<MLOD
**37**	2.005	<MLOD	<MLOD	<MLOD	1.238	<MLOD	<MLOD	<MLOD	<MLOD	<MLOD	<MLOD	<MLOD	<MLOD	<MLOD	0.233	<MLOD	<MLOD
**40**	<MLOD	<MLOD	<MLOD	<MLOD	<MLOD	<MLOD	0.050	<MLOD	<MLOD	<MLOD	<MLOD	<MLOD	<MLOD	<MLOD	<MLOD	<MLOD	<MLOD
**50**	1.748	<MLOD	<MLOD	<MLOD	<MLOD	<MLOD	<MLOD	<MLOD	<MLOD	<MLOD	<MLOD	<MLOD	<MLOD	<MLOD	<MLOD	<MLOD	<MLOD
**58**	<MLOD	<MLOD	<MLOD	0.563	0.175	<MLOD	<MLOD	<MLOD	<MLOD	<MLOD	<MLOD	<MLOD	<MLOD	<MLOD	<MLOD	<MLOD	<MLOD
**62**	0.438	<MLOD	<MLOD	<MLOD	<MLOD	<MLOD	<MLOD	<MLOD	<MLOD	<MLOD	<MLOD	<MLOD	0.928	0.025	<MLOD	0.019	<MLOD
**64**	3.391	<MLOD	<MLOD	<MLOD	<MLOD	<MLOD	0.245	<MLOD	<MLOD	<MLOD	<MLOD	<MLOD	<MLOD	<MLOD	<MLOD	<MLOD	<MLOD

**68**	2.044	<MLOD	<MLOD	<MLOD	<MLOD	<MLOD	<MLOD	<MLOD	<MLOD	<MLOD	<MLOD	<MLOD	<MLOD	<MLOD	<MLOD	<MLOD	<MLOD
**75**	1.499	<MLOD	<MLOD	0.494	0.133	<MLOD	<MLOD	<MLOD	<MLOD	<MLOD	<MLOD	<MLOD	<MLOD	<MLOD	<MLOD	<MLOD	<MLOD
**77**	1.050	<MLOD	<MLOD	<MLOD	<MLOD	<MLOD	<MLOD	<MLOD	<MLOD	<MLOD	<MLOD	<MLOD	<MLOD	<MLOD	<MLOD	<MLOD	<MLOD
**92**	0.193	<MLOD	<MLOD	<MLOD	<MLOD	<MLOD	<MLOD	<MLOD	<MLOD	<MLOD	<MLOD	<MLOD	0.601	<MLOD	<MLOD	<MLOD	<MLOD
**93**	3.617	<MLOD	<MLOD	<MLOD	0.137	<MLOD	<MLOD	<MLOD	<MLOD	<MLOD	<MLOD	<MLOD	<MLOD	<MLOD	<MLOD	<MLOD	<MLOD
**100**	0.430	<MLOD	<MLOD	<MLOD	<MLOD	<MLOD	<MLOD	<MLOD	<MLOD	<MLOD	<MLOD	<MLOD	<MLOD	<MLOD	<MLOD	<MLOD	<MLOD
**105**	<MLOD	<MLOD	<MLOD	1.249	<MLOD	0.456	<MLOD	<MLOD	<MLOD	<MLOD	<MLOD	<MLOD	<MLOD	<MLOD	<MLOD	<MLOD	<MLOD
**108**	1.520	<MLOD	<MLOD	<MLOD	<MLOD	<MLOD	<MLOD	<MLOD	<MLOD	<MLOD	<MLOD	<MLOD	<MLOD	<MLOD	<MLOD	<MLOD	<MLOD
**112**	1.823	<MLOD	<MLOD	1.393	<MLOD	<MLOD	<MLOD	<MLOD	<MLOD	<MLOD	<MLOD	<MLOD	<MLOD	<MLOD	<MLOD	<MLOD	<MLOD
**120**	<MLOD	<MLOD	<MLOD	0.480	<MLOD	<MLOD	<MLOD	<MLOD	<MLOD	<MLOD	<MLOD	<MLOD	<MLOD	<MLOD	<MLOD	<MLOD	<MLOD
**122**	<MLOD	<MLOD	<MLOD	<MLOD	<MLOD	<MLOD	<MLOD	<MLOD	<MLOD	<MLOD	<MLOD	<MLOD	<MLOD	<MLOD	<MLOD	<MLOD	<MLOD
**129**	<MLOD	<MLOD	<MLOD	<MLOD	<MLOD	<MLOD	0.053	<MLOD	<MLOD	<MLOD	<MLOD	<MLOD	<MLOD	<MLOD	<MLOD	<MLOD	<MLOD
**133**	1.871	<MLOD	<MLOD	<MLOD	<MLOD	<MLOD	<MLOD	<MLOD	<MLOD	<MLOD	<MLOD	<MLOD	<MLOD	<MLOD	<MLOD	<MLOD	<MLOD
**139**	0.289	<MLOD	<MLOD	<MLOD	2.316	<MLOD	<MLOD	0.503	<MLOQ	<MLOD	<MLOD	<MLOD	<MLOD	<MLOD	<MLOD	0.084	<MLOD
**147**	2.599	<MLOQ	<MLOD	<MLOD	2.895	<MLOD	<MLOD	<MLOD	<MLOD	<MLOD	<MLOD	<MLOD	<MLOD	<MLOD	<MLOD	<MLOD	<MLOD
**149**	<MLOD	<MLOD	<MLOD	2.536	1.121	<MLOD	<MLOD	<MLOD	<MLOD	<MLOD	<MLOD	<MLOD	<MLOD	<MLOD	<MLOD	<MLOD	<MLOD
**154**	0.496	0.278	<MLOD	<MLOD	<MLOD	<MLOD	<MLOD	<MLOD	<MLOD	<MLOD	<MLOD	<MLOD	<MLOD	<MLOD	<MLOD	<MLOD	<MLOD
**164**	0.611	<MLOD	<MLOD	<MLOD	<MLOD	<MLOD	<MLOD	<MLOD	<MLOD	<MLOD	<MLOD	<MLOD	<MLOD	<MLOD	<MLOD	<MLOD	0.680
**165**	<MLOD	<MLOD	<MLOD	<MLOD	<MLOD	<MLOD	<MLOD	<MLOD	<MLOD	<MLOD	<MLOD	<MLOD	<MLOD	<MLOD	<MLOD	<MLOD	0.482
**181**	1.460	<MLOD	<MLOD	<MLOD	<MLOD	<MLOD	<MLOD	<MLOD	<MLOD	<MLOD	<MLOD	<MLOD	<MLOD	<MLOD	<MLOD	<MLOD	<MLOD
**193**	0.198	<MLOD	<MLOD	<MLOD	<MLOD	<MLOD	0.410	<MLOD	<MLOD	<MLOD	<MLOD	<MLOD	0.724	<MLOD	<MLOD	<MLOD	<MLOD
**195**	0.758	<MLOD	<MLOD	<MLOD	<MLOD	<MLOD	<MLOD	<MLOD	<MLOD	<MLOD	<MLOD	<MLOD	<MLOD	<MLOD	<MLOD	<MLOD	<MLOD
**196**	0.180	<MLOD	<MLOD	<MLOD	<MLOD	<MLOD	0.312	<MLOD	<MLOD	<MLOD	<MLOD	<MLOD	<MLOD	<MLOD	<MLOD	<MLOD	<MLOD
**199**	4.651	<MLOD	<MLOD	0.744	<MLOD	<MLOD	0.228	<MLOD	0.384	<MLOD	<MLOD	<MLOD	<MLOD	<MLOD	<MLOD	<MLOD	<MLOD
**201**	<MLOD	<MLOD	<MLOD	0.263	<MLOD	<MLOD	0.232	<MLOD	<MLOD	<MLOD	<MLOD	<MLOD	<MLOD	<MLOD	<MLOD	<MLOD	<MLOD
**209**	0.471	<MLOD	<MLOD	<MLOD	<MLOD	<MLOD	2.264	0.735	<MLOD	<MLOD	<MLOD	<MLOD	<MLOD	<MLOD	<MLOD	0.091	<MLOD
**212**	0.434	<MLOD	<MLOD	<MLOD	<MLOD	<MLOD	<MLOD	0.634	<MLOD	<MLOD	<MLOD	<MLOD	<MLOD	<MLOD	<MLOD	<MLOD	<MLOD
**226**	0.214	<MLOD	<MLOD	<MLOD	<MLOD	<MLOD	<MLOD	<MLOD	<MLOD	<MLOD	<MLOD	<MLOD	<MLOD	<MLOD	<MLOD	<MLOD	<MLOD
**229**	0.368	<MLOD	<MLOD	<MLOD	<MLOD	<MLOD	0.856	<MLOD	<MLOD	<MLOD	<MLOD	<MLOD	<MLOD	<MLOD	<MLOD	<MLOD	<MLOD
**234**	<MLOD	<MLOD	<MLOD	<MLOD	<MLOD	<MLOD	0.111	<MLOD	<MLOD	<MLOD	<MLOD	<MLOD	<MLOD	<MLOD	<MLOD	<MLOD	<MLOD

**236**	0.440	<MLOD	<MLOD	<MLOD	<MLOD	<MLOD	1.060	<MLOD	<MLOD	<MLOD	<MLOQ	<MLOD	<MLOD	<MLOD	<MLOD	<MLOD	<MLOD
**245**	<MLOD	<MLOD	<MLOD	1.331	<MLOD	<MLOD	<MLOD	<MLOD	<MLOD	<MLOD	<MLOD	<MLOD	<MLOD	<MLOD	<MLOD	<MLOD	<MLOD
**248**	<MLOD	<MLOD	<MLOD	<MLOD	<MLOD	<MLOD	0.356	<MLOD	<MLOD	<MLOD	<MLOD	<MLOD	<MLOD	<MLOD	<MLOD	<MLOD	<MLOD
**253**	3.455	<MLOD	<MLOD	0.369	<MLOD	<MLOD	<MLOD	<MLOD	<MLOD	<MLOD	<MLOD	<MLOD	<MLOD	<MLOD	<MLOD	<MLOD	<MLOD
**254**	3.735	<MLOD	0.047	<MLOD	<MLOD	2.799	2.174	<MLOD	<MLOD	<MLOD	<MLOD	<MLOD	<MLOD	<MLOD	<MLOD	<MLOD	0.086
**257**	3.775	<MLOD	<MLOD	<MLOD	<MLOD	<MLOD	<MLOD	<MLOD	<MLOD	<MLOD	<MLOD	<MLOD	<MLOD	<MLOD	<MLOD	<MLOD	<MLOD
**260**	1.109	<MLOD	<MLOD	0.506	<MLOD	<MLOD	0.170	<MLOD	<MLOD	<MLOD	<MLOD	<MLOD	<MLOD	<MLOD	<MLOD	<MLOD	<MLOD
**261**	3.842	<MLOD	<MLOD	<MLOD	<MLOD	<MLOD	<MLOD	<MLOD	<MLOD	0.100	<MLOD	<MLOD	<MLOD	<MLOD	<MLOD	<MLOD	<MLOD
**280**	3.909	<MLOD	<MLOD	1.934	<MLOD	<MLOD	0.119	<MLOD	0.066	<MLOQ	<MLOD	<MLOD	<MLOD	<MLOD	<MLOD	<MLOD	<MLOD
**285**	<MLOD	<MLOD	<MLOD	<MLOD	<MLOD	<MLOD	<MLOD	<MLOD	<MLOD	<MLOD	<MLOD	<MLOD	0.600	<MLOD	<MLOD	<MLOD	<MLOD
**297**	5.950	<MLOD	<MLOD	<MLOD	<MLOD	<MLOD	<MLOD	<MLOD	<MLOD	<MLOD	<MLOD	<MLOD	<MLOD	<MLOD	<MLOD	<MLOD	<MLOD
**300**	2.881	<MLOD	<MLOD	1.066	<MLOD	<MLOD	<MLOD	<MLOD	<MLOD	<MLOD	<MLOD	<MLOD	<MLOD	<MLOD	<MLOD	<MLOD	<MLOD
**303**	0.324	<MLOD	<MLOD	<MLOD	1.976	1.780	1.950	<MLOD	<MLOD	<MLOD	<MLOD	<MLOD	1.663	<MLOD	<MLOD	<MLOD	0.263
**309**	<MLOD	<MLOD	<MLOD	<MLOD	<MLOD	<MLOD	0.045	<MLOD	<MLOD	<MLOD	<MLOD	<MLOD	<MLOD	<MLOD	<MLOD	<MLOD	<MLOD
**314**	<MLOD	<MLOD	<MLOD	<MLOD	<MLOD	<MLOD	0.275	<MLOD	0.416	<MLOD	<MLOD	0.099	<MLOD	<MLOD	<MLOD	<MLOD	<MLOD
**323**	0.438	<MLOD	<MLOD	<MLOD	<MLOD	<MLOD	<MLOD	0.551	<MLOD	<MLOD	<MLOD	<MLOD	<MLOD	<MLOD	<MLOD	<MLOD	<MLOD
**325**	1.109	<MLOD	<MLOD	<MLOD	<MLOD	<MLOD	<MLOD	<MLOD	<MLOD	<MLOD	<MLOD	<MLOD	3.274	<MLOD	<MLOD	<MLOD	<MLOD
**328**	<MLOD	<MLOD	<MLOD	<MLOD	<MLOD	<MLOD	0.067	<MLOD	<MLOD	<MLOD	<MLOD	<MLOD	<MLOD	<MLOD	<MLOD	<MLOD	<MLOD
**329**	<MLOD	<MLOD	<MLOD	0.921	<MLOD	<MLOD	0.124	<MLOD	<MLOD	<MLOD	<MLOD	<MLOD	<MLOD	<MLOD	<MLOD	<MLOD	<MLOD
**331**	0.717	<MLOD	<MLOD	<MLOD	<MLOD	<MLOD	<MLOD	0.629	<MLOD	<MLOD	<MLOD	<MLOD	1.976	<MLOD	<MLOD	<MLOD	<MLOD
**333**	<MLOD	<MLOD	<MLOD	<MLOD	<MLOD	<MLOD	<MLOD	<MLOD	<MLOD	<MLOD	<MLOD	<MLOD	<MLOD	<MLOD	<MLOD	<MLOD	<MLOD
**337**	1.587	<MLOD	<MLOD	<MLOD	<MLOD	<MLOD	<MLOD	<MLOD	<MLOD	<MLOD	<MLOD	<MLOD	<MLOD	<MLOD	<MLOD	<MLOD	<MLOD
**340**	0.442	<MLOD	<MLOD	<MLOD	<MLOD	<MLOD	<MLOD	<MLOD	<MLOD	<MLOD	<MLOD	<MLOD	<MLOD	<MLOD	<MLOD	<MLOD	<MLOD
**342**	<MLOD	<MLOD	<MLOD	<MLOD	<MLOD	<MLOD	0.321	<MLOD	<MLOD	<MLOD	<MLOD	<MLOD	0.674	<MLOD	<MLOD	<MLOD	0.551
**351**	3.842	<MLOD	<MLOD	1.647	<MLOD	<MLOD	0.132	<MLOD	<MLOD	<MLOD	<MLOD	0.117	<MLOD	<MLOD	<MLOD	<MLOD	<MLOD
**352**	0.486	<MLOD	<MLOD	<MLOD	<MLOD	<MLOD	<MLOD	0.427	<MLOD	<MLOD	<MLOD	<MLOD	<MLOD	0.029	<MLOD	<MLOD	<MLOD
**354**	0.378	<MLOD	<MLOD	<MLOD	<MLOD	<MLOD	<MLOD	<MLOD	<MLOD	<MLOD	<MLOD	<MLOD	<MLOD	<MLOD	<MLOD	<MLOD	<MLOD
**355**	<MLOD	<MLOD	<MLOD	<MLOD	0.997	<MLOD	<MLOD	<MLOD	<MLOD	<MLOD	<MLOD	<MLOD	<MLOD	<MLOD	<MLOD	<MLOD	<MLOD
**368**	<MLOD	<MLOD	<MLOD	0.463	<MLOD	<MLOD	<MLOD	<MLOD	0.229	<MLOD	<MLOD	<MLOD	<MLOD	<MLOD	<MLOD	<MLOD	<MLOD
**370**	3.321	<MLOD	<MLOD	0.561	0.156	<MLOD	<MLOD	<MLOD	<MLOD	<MLOD	<MLOD	<MLOD	<MLOD	<MLOD	<MLOD	<MLOD	<MLOD

**372**	4.861	<MLOD	<MLOD	0.518	<MLOD	<MLOD	<MLOD	<MLOD	<MLOD	<MLOD	<MLOD	<MLOD	<MLOD	<MLOD	<MLOD	<MLOD	<MLOD
**382**	1.523	<MLOD	<MLOD	1.619	0.156	<MLOD	<MLOD	<MLOD	<MLOD	<MLOD	<MLOD	<MLOD	<MLOD	<MLOD	<MLOD	<MLOD	<MLOD
**385**	0.456	<MLOD	<MLOD	0.803	<MLOD	<MLOD	<MLOD	<MLOD	<MLOD	<MLOD	<MLOD	<MLOD	<MLOD	<MLOD	<MLOD	<MLOD	<MLOD
**386**	0.578	<MLOD	<MLOD	<MLOD	<MLOD	<MLOD	<MLOD	<MLOD	0.334	<MLOD	<MLOD	<MLOD	<MLOD	<MLOD	<MLOD	<MLOD	<MLOD
**388**	<MLOD	<MLOD	<MLOD	0.515	<MLOD	<MLOD	<MLOD	<MLOD	<MLOD	<MLOD	<MLOD	<MLOD	<MLOD	<MLOD	<MLOD	<MLOD	<MLOD
**389**	<MLOD	<MLOD	<MLOD	0.336	<MLOD	<MLOD	<MLOD	<MLOD	0.128	<MLOD	<MLOD	0.140	<MLOD	<MLOD	<MLOD	<MLOD	<MLOD
**393**	<MLOD	<MLOD	<MLOD	1.467	1.175	<MLOD	<MLOD	0.451	0.089	<MLOD	<MLOD	<MLOD	<MLOD	<MLOD	0.361	<MLOD	<MLOD
**395**	<MLOD	<MLOD	<MLOD	<MLOD	<MLOD	<MLOD	0.200	<MLOD	<MLOD	<MLOD	<MLOD	<MLOD	<MLOD	<MLOD	<MLOD	<MLOD	<MLOD
**402**	<MLOD	<MLOD	<MLOD	0.270	<MLOD	<MLOD	<MLOD	<MLOD	<MLOD	<MLOD	<MLOD	<MLOD	<MLOD	<MLOD	<MLOD	<MLOD	<MLOD
**405**	0.729	<MLOD	<MLOD	1.899	<MLOD	<MLOD	<MLOD	<MLOD	<MLOD	<MLOD	<MLOD	<MLOD	<MLOD	<MLOD	<MLOD	<MLOD	<MLOD
**409**	1.317	<MLOD	<MLOD	<MLOD	<MLOD	<MLOD	<MLOD	<MLOD	<MLOD	<MLOD	<MLOD	<MLOD	<MLOD	<MLOD	<MLOD	<MLOD	<MLOD
**425**	<MLOD	<MLOD	<MLOD	<MLOD	<MLOD	<MLOD	0.119	<MLOD	<MLOD	<MLOD	<MLOD	<MLOD	<MLOD	<MLOD	<MLOD	<MLOD	<MLOD
**428**	1.236	<MLOD	<MLOD	<MLOD	<MLOD	<MLOD	<MLOD	<MLOD	<MLOD	<MLOD	<MLOD	<MLOD	<MLOD	<MLOD	<MLOD	<MLOD	<MLOD
**430**	4.723	<MLOD	<MLOD	0.485	<MLOD	<MLOD	<MLOD	<MLOD	<MLOD	<MLOD	<MLOD	<MLOD	<MLOD	<MLOD	<MLOD	<MLOD	<MLOD
**432**	0.701	0.282	<MLOD	<MLOD	1.897	<MLOD	<MLOD	<MLOD	<MLOD	<MLOD	<MLOD	<MLOD	<MLOD	<MLOD	<MLOD	<MLOD	<MLOD
**436**	<MLOD	<MLOD	<MLOD	2.461	0.178	<MLOD	<MLOD	<MLOD	<MLOD	<MLOD	<MLOD	<MLOD	<MLOD	<MLOD	<MLOD	<MLOD	<MLOD
**437**	0.637	<MLOD	<MLOD	<MLOD	<MLOD	<MLOD	<MLOD	<MLOD	<MLOD	<MLOD	<MLOD	<MLOD	<MLOD	<MLOD	<MLOD	<MLOD	<MLOD
**439**	<MLOD	<MLOD	<MLOD	<MLOD	<MLOD	<MLOD	<MLOD	<MLOD	<MLOD	<MLOD	<MLOD	<MLOD	0.521	<MLOD	<MLOD	<MLOD	<MLOD
**441**	<MLOD	<MLOD	<MLOD	1.565	<MLOD	<MLOD	<MLOD	<MLOD	<MLOD	<MLOD	<MLOD	<MLOD	<MLOD	<MLOD	<MLOD	<MLOD	<MLOD
**442**	0.535	<MLOD	<MLOD	<MLOD	<MLOD	<MLOD	<MLOD	<MLOD	<MLOD	<MLOD	<MLOD	<MLOD	<MLOD	<MLOD	<MLOD	<MLOD	<MLOD
**446**	1.890	<MLOD	<MLOD	<MLOD	<MLOD	<MLOD	<MLOD	<MLOD	<MLOD	<MLOD	<MLOD	<MLOD	<MLOD	<MLOD	<MLOD	<MLOD	<MLOD
**452**	0.442	<MLOD	<MLOD	0.531	<MLOD	<MLOD	0.176	<MLOD	<MLOD	<MLOD	<MLOD	0.097	<MLOD	<MLOD	<MLOD	<MLOD	<MLOD
**476**	0.180	<MLOD	<MLOD	<MLOD	<MLOD	<MLOD	0.319	<MLOD	<MLOD	<MLOD	0.169	0.852	<MLOD	<MLOD	<MLOD	<MLOD	<MLOD
**477**	<MLOD	0.111	<MLOD	<MLOD	<MLOD	<MLOD	<MLOD	<MLOD	<MLOD	<MLOD	<MLOD	<MLOD	<MLOD	<MLOD	<MLOD	<MLOD	0.173
**480**	2.396	<MLOD	<MLOD	<MLOD	<MLOD	<MLOD	<MLOD	<MLOD	<MLOD	<MLOD	<MLOD	<MLOD	<MLOD	<MLOD	<MLOD	<MLOD	<MLOD
**489**	<MLOD	<MLOD	<MLOD	<MLOD	<MLOD	<MLOD	<MLOD	<MLOD	<MLOD	<MLOD	<MLOD	<MLOD	<MLOD	<MLOD	<MLOD	<MLOD	<MLOD
**495**	1.174	<MLOD	<MLOD	<MLOD	<MLOD	<MLOD	<MLOD	<MLOD	<MLOD	<MLOD	<MLOD	<MLOD	<MLOD	<MLOD	<MLOD	<MLOD	<MLOD
**503**	0.784	<MLOD	<MLOD	<MLOD	<MLOD	<MLOD	<MLOD	<MLOD	<MLOD	<MLOD	<MLOD	<MLOD	<MLOD	<MLOD	<MLOD	<MLOD	<MLOD
**506**	1.032	<MLOD	<MLOD	0.415	<MLOD	<MLOD	<MLOD	<MLOD	<MLOD	<MLOD	<MLOD	<MLOD	<MLOD	<MLOD	<MLOD	<MLOD	<MLOD
**510**	0.937	<MLOD	<MLOD	0.974	<MLOD	<MLOD	<MLOD	<MLOD	<MLOD	<MLOD	<MLOD	<MLOD	<MLOD	<MLOD	<MLOD	<MLOD	<MLOD

**512**	<MLOD	<MLOD	<MLOD	<MLOD	<MLOD	<MLOD	0.194	<MLOD	<MLOD	<MLOD	<MLOD	<MLOD	<MLOD	<MLOD	<MLOD	<MLOD	<MLOD
**513**	2.914	<MLOD	<MLOD	<MLOD	0.276	<MLOD	<MLOD	<MLOD	<MLOD	<MLOD	<MLOD	<MLOD	0.975	<MLOD	<MLOD	<MLOD	<MLOD
**514**	<MLOD	<MLOD	<MLOD	<MLOD	0.217	<MLOD	<MLOD	<MLOD	<MLOD	<MLOD	<MLOD	<MLOD	<MLOD	<MLOD	<MLOD	<MLOD	<MLOD
**517**	4.061	<MLOD	<MLOD	0.314	0.443	0.300	<MLOD	<MLOD	<MLOD	<MLOD	<MLOD	<MLOD	<MLOD	<MLOD	<MLOD	<MLOD	<MLOD
**518**	<MLOD	<MLOD	<MLOD	2.474	<MLOD	<MLOD	<MLOD	<MLOD	<MLOD	<MLOD	<MLOD	<MLOD	<MLOD	<MLOD	<MLOD	<MLOD	<MLOD
**521**	3.142	<MLOD	<MLOD	0.835	0.123	<MLOD	<MLOD	<MLOD	<MLOD	<MLOD	<MLOD	<MLOD	<MLOD	<MLOD	<MLOD	<MLOD	<MLOD
**522**	1.272	<MLOD	<MLOD	<MLOD	<MLOD	<MLOD	<MLOD	<MLOD	<MLOD	0.079	<MLOD	<MLOD	<MLOD	<MLOD	<MLOD	<MLOD	<MLOD
**523**	<MLOQ	<MLOD	<MLOD	<MLOD	<MLOD	<MLOD	<MLOD	<MLOD	0.063	<MLOD	<MLOD	0.098	<MLOD	<MLOD	<MLOD	<MLOD	<MLOD
**528**	0.226	<MLOD	<MLOD	<MLOD	<MLOD	<MLOD	0.294	<MLOD	<MLOD	<MLOD	<MLOD	<MLOD	<MLOD	<MLOD	<MLOD	<MLOD	<MLOD
**533**	<MLOD	<MLOD	<MLOD	2.032	<MLOD	<MLOD	<MLOD	<MLOD	<MLOD	<MLOD	<MLOD	<MLOD	<MLOD	<MLOD	<MLOD	<MLOD	<MLOD
**534**	<MLOD	<MLOD	<MLOD	1.261	<MLOD	<MLOD	<MLOD	<MLOD	<MLOD	<MLOD	<MLOD	<MLOD	<MLOD	<MLOD	<MLOD	<MLOD	<MLOD
**539**	3.077	<MLOD	<MLOD	4.280	1.143	<MLOD	<MLOD	<MLOD	<MLOD	<MLOD	<MLOD	<MLOD	<MLOD	<MLOD	<MLOD	<MLOD	<MLOD
**548**	4.189	<MLOD	<MLOD	<MLOD	<MLOD	<MLOD	0.406	<MLOD	<MLOD	<MLOD	<MLOD	<MLOD	<MLOD	<MLOD	<MLOD	<MLOD	<MLOD
**555**	0.481	<MLOD	<MLOD	<MLOD	<MLOD	<MLOD	<MLOD	0.489	<MLOD	<MLOD	<MLOD	<MLOD	<MLOD	0.121	0.244	0.284	<MLOD
**560**	2.544	<MLOD	<MLOD	<MLOD	<MLOD	<MLOD	<MLOD	<MLOD	<MLOD	<MLOD	<MLOD	<MLOD	<MLOD	<MLOD	<MLOD	<MLOD	<MLOD
**561**	<MLOD	<MLOD	<MLOD	<MLOD	<MLOD	<MLOD	0.162	<MLOD	<MLOD	<MLOD	<MLOD	<MLOD	<MLOD	<MLOD	<MLOD	<MLOD	<MLOD
**562**	<MLOD	<MLOD	<MLOD	1.381	<MLOQ	<MLOD	<MLOD	<MLOD	<MLOD	<MLOD	<MLOD	<MLOD	<MLOD	<MLOD	<MLOD	<MLOD	<MLOD
**571**	0.474	0.254	<MLOD	<MLOD	<MLOD	<MLOD	<MLOD	<MLOD	<MLOD	<MLOD	<MLOD	<MLOD	<MLOD	<MLOD	<MLOD	<MLOD	<MLOD
**574**	0.590	<MLOD	<MLOD	<MLOD	<MLOD	<MLOD	<MLOD	<MLOD	<MLOD	<MLOD	<MLOD	<MLOD	<MLOD	<MLOD	0.211	<MLOD	<MLOD
**577**	<MLOD	<MLOD	<MLOD	<MLOD	<MLOD	<MLOD	0.133	<MLOD	<MLOD	<MLOD	<MLOD	<MLOD	<MLOD	<MLOD	<MLOD	<MLOD	<MLOD
**582**	0.260	<MLOD	<MLOD	<MLOD	<MLOD	<MLOD	<MLOD	<MLOD	<MLOD	<MLOD	<MLOD	<MLOD	<MLOD	<MLOD	<MLOD	<MLOD	<MLOD
**586**	0.387	<MLOD	<MLOD	<MLOD	<MLOD	<MLOD	0.170	<MLOD	<MLOD	0.060	<MLOD	<MLOD	<MLOD	<MLOD	<MLOD	<MLOD	<MLOD
**587**	<MLOD	<MLOD	<MLOD	<MLOD	<MLOD	<MLOD	<MLOD	<MLOD	<MLOD	<MLOD	<MLOD	<MLOD	<MLOD	<MLOD	<MLOD	<MLOD	<MLOD
**590**	0.345	<MLOD	<MLOD	1.048	<MLOD	<MLOD	0.281	<MLOD	0.337	<MLOD	<MLOD	0.114	<MLOD	<MLOD	<MLOD	<MLOD	<MLOD

Table S2. List of Internal standards used as surrogate standards.

	**Compounds**	**Acronyms**	**Manufacturer. Product Code. *Mass-Labelled Standard. Mass-Labelled Product Code**	**Obs**
1	Trifluorocarboxylic acid	TFA	Toronto Research Chemicals Inc. (13C2-TFA)	
2	Perfluoropropionic acid	PFPrA	Wellington. 13C3-PFPrA	n.d.
3	Perfluorobutanoic acid	PFBA	Wellington. PFAC300719. MPFBA (13C4)	
4	Perfluoropentanoic acid	PFPeA	Wellington. PFAC300719. M5PFPeA (13C5).	
5	Perfluorohexanoic acid	PFHxA	Wellington. PFAC300719. M5PFHxA (1.2.3.4.5-13C5)	
6	Perfluoroheptanoic acid	PFHpA	Wellington. PFAC300719. M4PFHpA (1.2.3.4-13C4)	
7	Perfluorooctanoic acid	PFOA	Wellington. PFAC300719. M8PFOA (13C8)	
8	Perfluorononanoic acid	PFNA	Wellington. PFAC300719. M9PFNA (13C9).	
9	Perfluorodecanoic acid	PFDA	Wellington. PFAC300719. M6PFDA (1.2.3.4.5.6-13C6)	
10	Perfluoroundecanoic acid	PFUnDA	Wellington. PFAC300719. M7PFUdA (1.2.3.4.5.6.713C7)	
11	Perfluorododecanoic acid	PFDoDA	Wellington. PFAC300719. MPFDoA (1.2-13C2).	n.d.
12	Perfluorotetradecanoic acid	PFTeDA	Wellington 13C2-PFTeDA	n.d.
13	Perfluoromethanesulfonic acid	PFMS	Wellington 13C-PFMS	
14	Perfluoroethanesulfonic acid	PFEtS	Identification with native external standard and quantification with 13C3-PFBS	n.d.
15	Perfluoropropionicsulfonic acid	PFPrS	Identification by native standard<br>Internal standard 13C3-PFB	n.d.
16	Perfluobutanesulfonic acid	PFBS	Wellington. PFAC300719. M3PFBS (2.3.4-13C3)	
17	Perfluoropentane sulfonic acid	PFPeS	Wellington. PFAC300719. *d5-n-EtFOSAA (MPFAC24ES0218)	
18	Perfluorohexane sulfonic acid	PFHxS	Wellington. PFAC300719. M3PFHxS (1.2.3-13C3	
19	Perfluoroheptane sulfonic acid	PFHpS	Wellington 13C3-PFHpS	
20	Perfluorooctane sulfonic acid	PFOS	Wellington. PFAC300719. M8PFOS (13C8)).	
21	Perfluorononanesulfonic acid	PFNS	Wellington. PFAC300719. *M8PFOS (MPFAC24ES0218)	n.d.
22	Perfluorooctane sulfonamide	FOSA	Wellington: M8FOSA-I. Perfluoro-1-(13C8) octanesulfonamide Solution	n.d.
23	N-methyl perfluorooctane sulfonamidoacetic acid	N-MeFOSAA	Wellington: d3-N-MeFOSAA	n.d.
24	N-ethyl perfluorooctane sulfonamidoacetic acid	N-EtFOSAA	Wellington: d5-N-EtFOSAA	n.d.
25	4:2 Fluorotelomer sulfonic acid	4:2 FTS	Wellington:13C2-4:2FTS	n.d.
26	6:2 Fluorotelomer sulfonic acid	6:2 FTS	Wellington:13C2-6:2FTS	n.d.
27	8:2 Fluorotelomer sulfonic acid	8:2 FTS	Wellington:13C2-8:2FTS	n.d.
28	4,8-dioxa-3H-perfluorononanoic acid	Adona	Wellington: 13C3-ADONA	
29	hexafluoropropylene oxide dimer acid (HFPO-DA)	Gen-X	Wellington: 13C3-HFPO-DA	

n.d. or quantified in the samples.

Table S3. Quality assurance and quality control parameters of the analytical method.

Compound	Chemical structure	CAS	MLOD (ng/ml)	MLOQ (ng/ml)	%Recovery 10 ng/l	Precision (%RSD)* Intra	Inter
Trifluoro carboxylic acid (TFA)	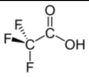	76-05-1	0.055	0.172	82	11.6	15.1
Perfluoropropionic acid (PFPrA)	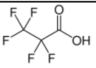	422-64-0	0.020	0.675	70	20.1	36.2
Perfluorobutanoic acid (PFBA)	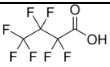	375-22-4	0.002	0.007	74	5.7	17.9
Perfluoropentanoic acid (PFPeA)	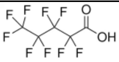		0.002	0.007	88	4.4	23.1
Perfluorohexanoic acid (PFHxA)	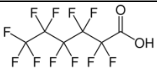	307-24-4	0.006	0.017	92	7.2	23.2
Perfluoroheptanoic acid (PFHpA)	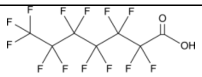	375-85-9	0.014	0.045	92	6.6	20.5
Perfluorooctanoic acid (PFOA)	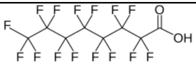	335-67-1	0.008	0.024	91	4.0	23.9
Perfluorononanoic acid (PFNA)	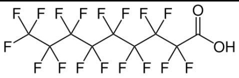	375-95-1	0.002	0.007	90	5.7	25.9
Perfluorodecanoic acid (PFDA)	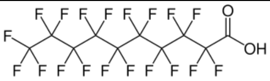	2706-90-3	0.002	0.007	80	7.0	36.4
Perfluoroundecanoic acid (PFUnDA)	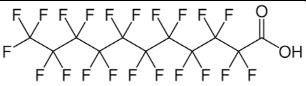	2058-94-8	0.004	0.014	77	15.4	32.8
Perfluorododecanoic acid (PFDoDA)	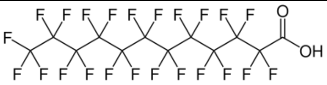	307-55-1	0.002	0.006	70	14	31.4
Perfluoromethanesulfonate (PFMS)	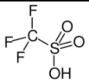	1493-13-6	0.006	0.017	98	10.5	25.8
Perfluoroethanesulfonic acid (PFEtS)	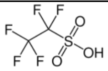	354-88-1	0.700	2.00	77	6.8	20.3
Perfluoropropionicsulfonic acid (PFPrS)	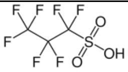	423-41-6	0.121	0.600	80	16.2	31.7
Perfluobutanesulfonic acid (PFBS)	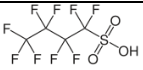	375-73-5	0.013	0.041	95	1.4	15.6
Perfluoropentane sulfonic acid **(**PFPeS)	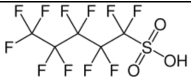	2706-91-4	0.045	0.140	68	17.7	32.5
Perfluorohexane sulfonic acid (PFHxS)	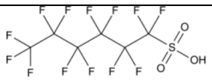	355-46-4	0.002	0.007	105	10.1	29.2
Perfluorooctane sulfonic acid (PFOS)	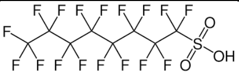	1763-23-1	0.044	0.137	91	12.0	32.8
Perfluorononanesulfonic acid (PFNS)	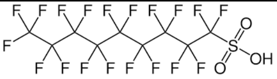	68259-12-1	0.007	0.021	52	0.8	14.3
4.8-Dioxa-3H perfluorononanoic acid (ADONA)	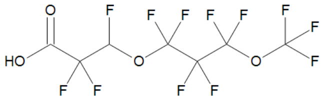	919005-14-4	0.003	0.013	102	12.5	21.3
HFPO-DAT (GenX)	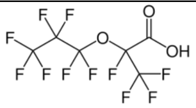	13252-13-6	0.008	0.024	111	10.3	17.3

Chemical Abstracts Service (CAS); Method limit of detection (MLOD); Method limit of quantification (MLOQ); *Precision Interday n=3. Intraday n=3
